# Therapeutic hypothermia in patients with traumatic brain injury: an umbrella review

**DOI:** 10.1186/s12883-025-04463-3

**Published:** 2025-10-24

**Authors:** Hamidreza Ashayeri, Moloud Balafar, Amir Ebrahimi, Hassan Soleimanpour, Nasim Hajipoor-Kashgsaray, Kavous Shahsavarinia, Hanieh Salehi-Pourmehr

**Affiliations:** 1 Research Center for Evidence-based Medicine, Iranian EBM Centre: A JBI Centre of Excellence, Faculty of Medicine, Tabriz University of Medical Sciences, Tabriz, Iran; 2 Student Research Committee, Tabriz University of Medical Sciences, Tabriz, Iran; 3 Emergency and Trauma Care Research Center, Tabriz University of Medical Sciences, Tabriz, Iran; 4 Medical Philosophy and History Research Center, Tabriz University of Medical Sciences, Tabriz, Iran; 5 Tuberculosis and Lung Disease Research Center, Tabriz University of Medical Sciences, Tabriz, Iran

**Keywords:** Therapeutic hypothermia, Mortality, Morbidity, Traumatic brain injury, Pneumonia

## Abstract

**Background:**

Therapeutic hypothermia (TH) is an intervention conducted to reduce brain damage in neonatal asphyxia and postcardiac arrest patients. Traumatic brain injury (TBI) dysregulates cerebral blood flow and causes ischemic brain damage. TH could lower brain damage by reducing the metabolism rate and need for oxygen. In this umbrella review, we aim to investigate different aspects of TH intervention in TBI patients.

**Methods:**

We searched MEDLINE, Scopus, Embase, Cinahl, Epistemonikos, Cochrane Library, Web of Science, and Google Scholar for relevant systematic reviews until August 03, 2025. Then, studies entered the screening process, and systematic reviews of TH`s effect on TBI patients were included. We excluded studies without any quality assessments. Data for mortality, favorable and unfavorable neurologic outcomes, and possible complications of TH were extracted from the included studies. We avoided meta-analysis due to the vast heterogeneity between the studies’ methodologies.

**Results:**

Of the 1466 studies identified from the primary search, 30 met our inclusion criteria. According to the JBI critical appraisal tool for systematic reviews, 29 studies were of high quality, and 1 was of medium quality. Studies had different methodologies, such as different induction methods and durations, different age groups, target temperatures, and rewarming rates. Mortality was the most common outcome assessed by the studies. Morbidity was assessed as either favorable or unfavorable neurological outcomes at a follow-up of 1,3,6,12,24 months. Pneumonia was the most investigated side-effect of TH, and most studies reported a greater chance of pneumonia in the TH group. Other investigated complications of TH were electrolyte abnormalities, coagulopathy, and arrhythmia.

**Conclusion:**

Studies have shown controversial results regarding the effect of TH on mortality and morbidity. Differences in the target population, hypothermia protocol, and quality of included studies may be responsible for part of this controversy. Some of the important parameters that may affect the results are the age of TBI patients, the use of barbiturates, target TH temperature, rewarming rates, and method of cooling. Additionally, the subgroup analysis of high-quality studies differed from the pooled analysis provided by each study. More high-quality studies with specific protocols are needed to better understand TH’s role in TBI patients.

**Supplementary Information:**

The online version contains supplementary material available at 10.1186/s12883-025-04463-3.

## Background

Traumatic brain injury (TBI) is defined as a brain injury caused by traumatic events that affect the brain’s normal function [1]. In 2017, the global annual burden of TBI was estimated to be 400 billion US$ [2]. The Glasgow Coma Score (GCS) of patients determines the severity of TBI. TBI with a GCS of 13–15 is considered mild TBI, those with a GCS of 9–12 are considered moderate TBI, and those with a GCS < 9 are considered severe TBI [3]. An increase in brain metabolism due to glutamate secretion is noted after TBI. Increased cellular metabolism increases oxidative stress on neurons, causing cell death. Cell damage can also be increased by other means, such as hemorrhage and herniation [4, 5]. Starting treatment within the first hour after the injury for severe TBI patients is crucial since it reduces mortality and disability [6].

Hypothermia has been proposed to have a neuroprotective effect by lowering neural activity, rate of metabolism, and the inflammatory response [7]. Many systematic reviews have investigated the role of therapeutic hypothermia (TH) in the acute management of TBI patients. However, the data regarding the hypothermia-related exercise outcomes in TBI patients are controversial. Additionaly, there are essential differences between them, such as the included population, use of prophylactic hypothermia, targeted temperature, and duration of hypothermia. In this umbrella review, we aimed to evaluate the quality of evidence and summarize the results of systematic reviews and meta-analyses to better apply the therapeutic hypothermia protocol in managing TBI.

## Method

### Umbrella review methods

The umbrella review aims to gather, organize, and evaluate data from existing systematic reviews about a particular research question [8]. Joanna Briggs Institute (JBI) instructions were used to conduct an umbrella review. After a systematic search, systematic reviews (with or without meta-analysis) exploring the use and outcome of TH in the management of TBI and side effects in TBI patients were included. The research question and PICO of our studies are as follows:

- Review question: What is the effectiveness of therapeutic hypothermia on mortality and neurological outcomes in patients with traumatic brain injury?
- Types of participants (P): This review included systematic reviews of traumatic brain injury (TBI) patients with a focus on therapeutic hypothermia (TH). Participants of any age, demographic background, or sociocultural status were eligible, with no restriction on disease duration or comorbidities.
- Intervention (I): This umbrella review included systematic reviews that evaluated the effectiveness of TH on TBI patient outcomes.
- Comparison (C): This review considered studies that used other therapeutic approaches for the management of TBI.
- Outcomes (O): This umbrella review assessed intraoperative and perioperative outcomes, including mortality, morbidity, neurological outcomes, pneumonia, and other complications such as arrhythmia or hypokalemia.

### Literature search

We searched MEDLINE, CINAHL, Epistemonikos, the Cochrane Library, Scopus, Web of Science, and Embase to find relevant systematic reviews in English on January 08, 2024, and updated the search on August 03, 2025. We also conducted a manual search on 30 June 2024 in Google Scholar for newly published studies. The following keywords were used to search the databases: Brain Trauma, Craniocerebral Trauma, Skull Trauma, Parietal Trauma, Occipital trauma, Forehead trauma, Frontal Trauma, Brain Injuries, Hypothermia, Traumatic Brain Injury, TBI, Head trauma, and Hypothermia. A systematic review filter was used in the respective databases. There were no other restrictions regarding the time of publication, language, or country of origin. The full search query of each database is available in Supplementary File 1.

### Inclusion criteria

We included the latest and updated versions of systematic reviews of clinical trials and observational studies that compared the therapeutic (mortality, morbidity, and neurological outcome after TBI) or adverse effects (e.g., pneumonia, hypokalemia, arrhythmia, and bleeding) of TH as an intervention method between the treatment group and the control group. Only studies in English were included, and there was no restriction regarding the population characteristics, country of origin, or hypothermia protocol used. Exclusion criteria were as follows: narrative reviews, cohort studies, case-control studies, cross-sectional studies, book chapters, case reports & series, conference abstracts, studies lacking controls, quality assessment or risk of bias reports, studies with an available updated version, and studies not in English. Three authors separately screened the selected studies’ titles, abstracts, and full texts. Then, the full text was evaluated for eligibility. In cases of disagreement, a Fourth author was consulted.

### Data extraction

Two authors participated in the data extraction. The extracted data included general data (first named author, country, and year of publication) and demographic data (types and number of included studies, total individuals involved, and method of quality assessment), the outcome, complications, and methods of TH intervention (target temperature, duration, and rewarming rate).

### Assessment of methodological quality of included studies and quality of evidence

Two authors used JBI critical appraisal tools for systematic review [9] to appraise studies in mythology (search strategies, selected resources, the inclusion of studies, quality assessment by each study, etc.), methods used to minimize bias or calculate the likelihood of possible publication bias, and consistency of results with recommended practices.

### GRADE assessment

The GRADE (Grading of Recommendations, Assessment, Development, and Evaluations) framework was employed to assess the quality of evidence for the outcomes reported in this umbrella review. The GRADE approach evaluates evidence based on five domains and considers three factors that may increase the quality of evidence: large effect size, dose-response gradient, and plausible confounding. Two authors conducted the assessment independently, with disagreements resolved through discussion or consultation with a third reviewer.

#### Outcomes assessed

The following key outcomes were evaluated using GRADE:

1. Mortality: Defined as the incidence of death in patients with traumatic brain injury (TBI) treated with therapeutic hypothermia (TH) versus normothermia.
2. Neurological Outcomes: Categorized as favorable (e.g., Glasgow Outcome Scale [GOS] 4–5) or unfavorable (e.g., GOS ≤ 3) at follow-up periods of 1, 3, 6, 12, and 24 months.
3. Complications: Including pneumonia, arrhythmia, coagulopathy, and electrolyte abnormalities.

#### GRADE criteria

1. Risk of Bias: Evaluated using the JBI critical appraisal tool for systematic reviews. Studies with high methodological quality (e.g., adequate randomization, allocation concealment, blinding) were prioritized.
2. Inconsistency: Assessed by examining heterogeneity (I² statistic) in meta-analyses. High inconsistency (I² > 50%) downgraded the evidence quality.
3. Indirectness: Considered whether the included studies directly addressed the research question (e.g., population, intervention, comparator, outcomes).
4. Imprecision: Evaluated based on confidence intervals (CIs) and sample sizes. Wide CIs or small sample sizes led to downgrading.
5. Publication Bias: Assessed using funnel plots or qualitative evaluation if fewer than 10 studies were included.

#### Data synthesis

- Evidence from systematic reviews and meta-analyses was categorized as high, moderate, low, or very low quality.
- Subgroup analyses (e.g., age, TH duration, target temperature) were considered to explain heterogeneity.

### Data synthesis and method of analysis

The studies’ data are presented in a table according to the JBI method for umbrella reviews. Supplementary File 2 summarizes the different parameters involved in therapeutic hypothermia intervention based on the PICO process. The studies differed in the induction time, duration, use of TH as a prophylactic or treatment therapy, target temperature, and methods of applying TH. Because of these and the high calculated overlap, we didn`t perform any meta-analysis.

The covered area (CA) and corrected covered area (CCA) formulas provided by Pieper et al. [10] are used in overview studies to define the overlap of primary studies between systematic reviews. Overlap was defined as the number of primary articles that were repeated in more than one meta-analysis.

Covered Area (CA): $\:\frac{N}{rc}$, Corrected Covered Area (CCA): $\:\frac{N-r}{rc-r}$

N: The sum of primary published studies and repeated studies is counted to calculate N.

r: Number of rows or index publications.

c: Number of columns or reviews.

We have determined the overlap (CA and CCA) between studies of each section by using the GROOVE tool [11] and provided the graphical representation of overlap.

## Results

**Fig. 1:**
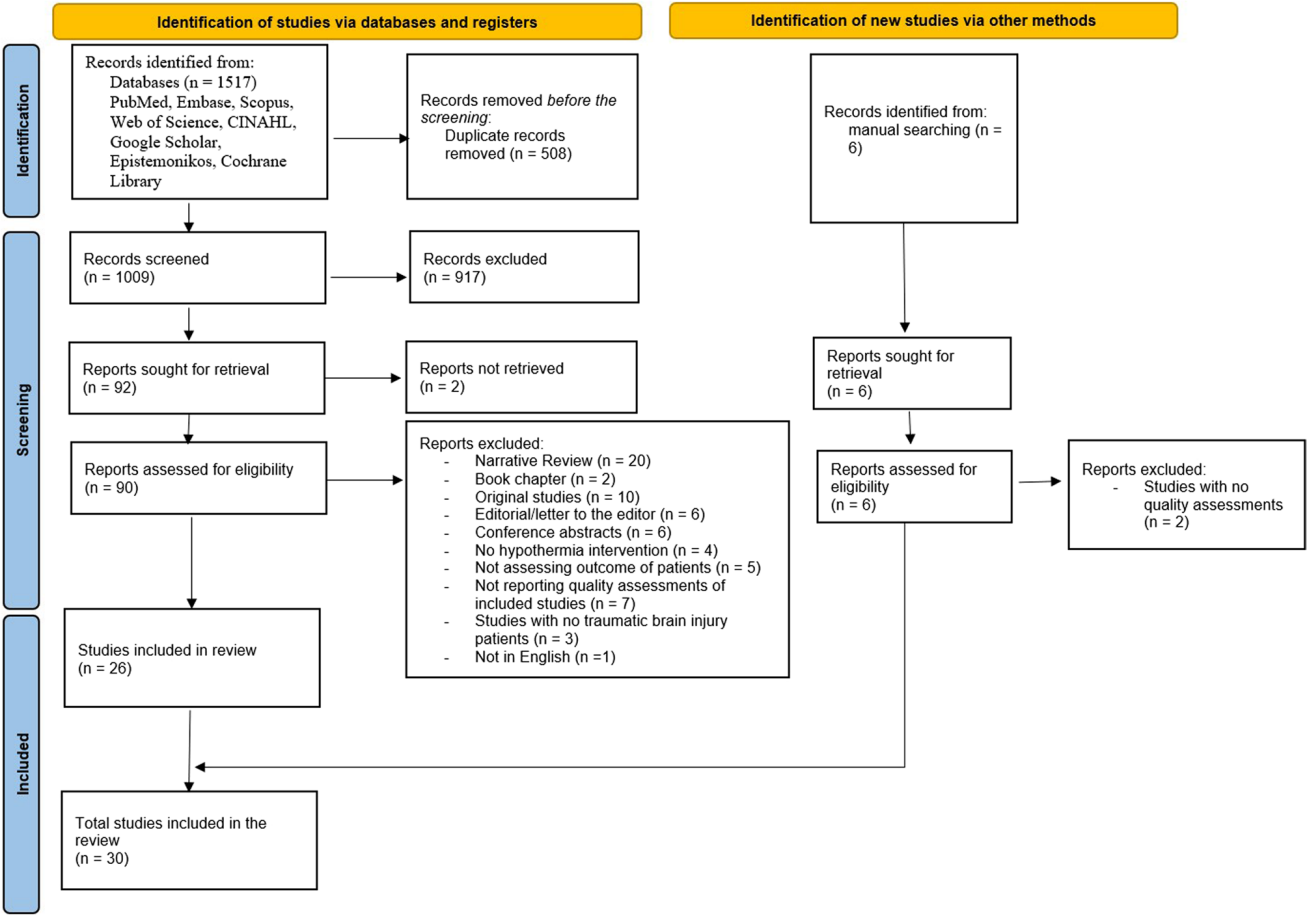
PRISMA flow diagram

A primary search of the databases resulted in 1517 studies, and after removing duplicate records, 1009 studies remained. These records were screened. After excluding studies that didn`t meet the eligibility criteria, 26 studies were included. Four studies were found via a manual search in Google Scholar. The PRISMA diagram in Fig. 1 summarizes this umbrella review’s search results and selection process.

### Study characteristics

We included 30 systematic reviews from 2002 to 2023 [12–41]. Most reviews included only randomized trials or randomized controlled trials (RCTs). Two studies included clinical trials [34, 40], and one included RCTs and retrospective quasi-experimental studies [15]. The sample size ranged between 25 and 3909 TBI patients. A Glasgow outcome scale (GOS) ≤ 3 was considered an unfavorable neurologic outcome in most of the reviews. Although the studies differed, they all had a hypothermia duration of > 12 h (mostly more than 24 h). The target temperature ranged between 30 and 36 °C. The rewarming rate was reported in 19 studies [12–16, 19, 20, 22, 24, 26, 27, 29, 32–34, 36–38, 41]. The characteristics of the included studies and a summary of the results of the meta-analysis are also reported in supplementary file 2.

### Quality assessment

A summary of the quality assessment of the included studies is presented in Supplementary File 3. The JBI critical appraisal tool for systematic reviews and research syntheses assesses quality based on eleven criteria. The majority were assessed as high quality, and no studies were rated as low or very low quality. This indicates that the body of evidence informing the review’s conclusions is generally robust and of a high standard.

The studies by Dunkley et al. [15], Harris et al. (2002) [20], Henderson et al. [21], McIntyre et al. [22], Leng et al. [34], and Du et al. [25] were all marked “Unclear” for the third criterion. Another notable weakness across a subset of the reviews was the failure to assess the likelihood of publication bias (the ninth criterion). The most common area of uncertainty was whether the reported data supported the review’s recommendations for policy and/or practice. A significant majority of the studies (28 out of 30) were marked as “Unclear” for this criterion—only two studies, by Peterson et al. [26] and Harris et al. (2012) [41] had a “Yes” rating for Q10. The identified weaknesses in specific quality domains, such as the assessment of publication bias and the support for recommendations, highlight areas for improvement in future systematic reviews on this topic.

#### GRADE assessment findings

For the critical outcome of mortality, we found moderate-quality evidence supporting TH in TBI. While most included systematic reviews demonstrated high methodological quality (29/30), some underlying RCTs had limitations in allocation concealment and blinding procedures. We observed moderate heterogeneity across studies, primarily attributable to variations in TH protocols regarding duration and target temperatures. The evidence showed minimal indirectness as studies directly compared TH with normothermia in comparable TBI populations. While large meta-analyses yielded precise estimates, certain subgroup analyses exhibited wider confidence intervals. Funnel plot analysis suggested a low likelihood of publication bias for this outcome.

Regarding neurological outcomes, the evidence quality differed between endpoints. For favorable outcomes, we rated the evidence as low quality due to unblinded outcome assessment in some studies, substantial heterogeneity from variable follow-up durations, and imprecise effect estimates in subgroups. In contrast, evidence for reducing unfavorable outcomes was of moderate quality, supported by consistent findings across high-quality reviews with explainable heterogeneity related to cooling duration and rewarming protocols.

Complication profiles demonstrated variable evidence quality. We assessed pneumonia risk as moderate-quality evidence, with consistent findings across studies showing increased risk. Arrhythmia evidence was rated as low quality due to methodological variability in detection and small sample sizes, leading to imprecise estimates. Coagulopathy and electrolyte abnormalities had very low-quality evidence, limited by sparse data from small RCTs and indirect outcome measures. Table 1 summarizes the results of the GRADE assessment.

**Table 1:** GRADE summary of findings for therapeutic hypothermia in traumatic brain injury

Outcome	Relative Effect (95% CI)	Participants (Studies)	Quality (GRADE)	GRADE Domains (↓/↑ Reasons)
Mortality				
Overall	RR 0.81–1.26	3,909 (32 RCTs)	⨁⨁⨁◯ Moderate	- ↓ Risk of bias: Some RCTs had unclear allocation concealment - ↓ Inconsistency: I²=45% (protocol variations) - ↑ Dose-response: Stronger effect with longer cooling duration
Prophylactic TH	RR 0.84 (0.72–0.98)	1,827 (19 RCTs)	⨁⨁⨁◯ Moderate	- ↓ Imprecision: Wide CI in subgroup analyses - ↑ Large effect: RR 0.63 for systemic cooling
Pediatric TBI	RR 1.73 (1.06–2.84)	366 (6 RCTs)	⨁⨁◯◯ Low	- ↓↓ Risk of bias: Unblinded outcome assessment - ↓ Inconsistency: I²=68%
Neurological Outcomes				
Favorable (GOS 4–5)	RR 0.89–1.56	2,523 (15 RCTs)	⨁⨁◯◯ Low	- ↓↓ Inconsistency: I²=60% - ↓ Imprecision: Wide CIs
Unfavorable (GOS ≤ 3)	RR 0.77 (0.67–0.88)	3,110 (37 RCTs)	⨁⨁⨁◯ Moderate	- ↓ Publication bias: Funnel plot asymmetry - ↑ Consistency: I²=40%
Complications				
Pneumonia	RR 1.51 (1.12–2.03)	1,617 (13 RCTs)	⨁⨁⨁◯ Moderate	- ↓ Indirectness: Variable pneumonia definitions - ↑ Precision: Narrow CI
Arrhythmia	RR 1.75 (1.14–2.70)	442 (7 RCTs)	⨁⨁◯◯ Low	- ↓↓ Risk of bias: Unsystematic ECG monitoring - ↓ Imprecision: Small sample
Coagulopathy	OR 2.22 (1.73–2.71)*	90 (2 RCTs)	⨁◯◯◯ Very Low	- ↓↓↓ Imprecision: Single study - ↓↓ Indirectness: Lab values only

Downgrade Reasons (↓):

Risk of bias: Unblinded outcome assessment, unclear randomization

Inconsistency: High heterogeneity (I²>50%) due to protocol variations

Indirectness: Population/intervention differences (e.g., pediatric vs. adult TBI)

Imprecision: Wide confidence intervals or small sample sizes

Publication bias: Funnel plot asymmetry or industry-funded studies

Upgrade Reasons (↑):

Large effect: RR <0.5 or >2 in consistent studies

Dose-response: Stronger effects with longer cooling durations

Plausible confounding: Bias would reduce the observed effect

*TH* Therapeutic Hypothermia, *TBI* Traumatic Brain Injury, *GOS* Glasgow Outcome Scale

*Partial thromboplastin time prolongation (no clinical bleeding reported)

### Mortality

**Fig. 2:**
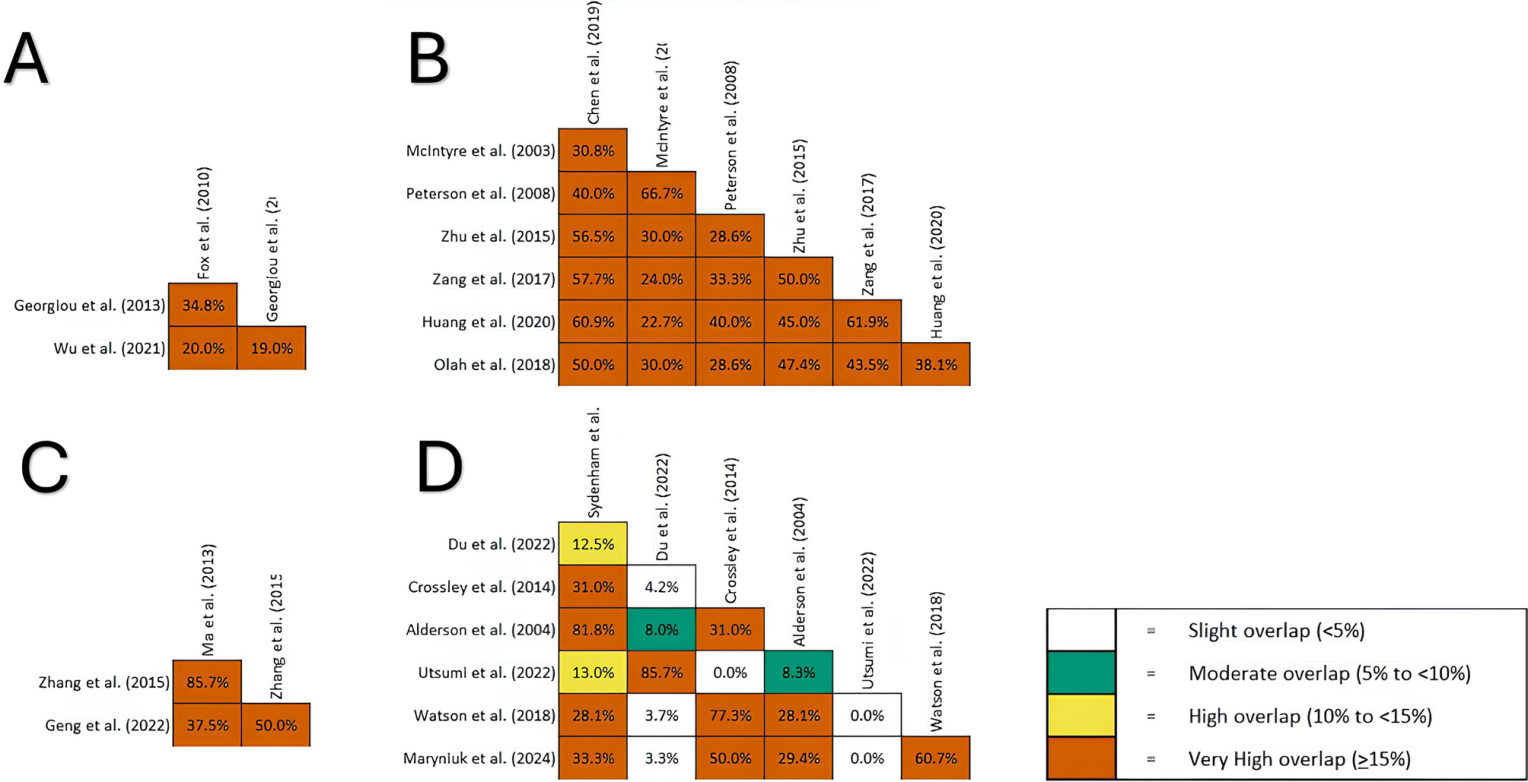
Graphical representation of the overlap between the studies investigating effects of prophylactic hypothermia (**A**), TH in adults (**B**), children (**C**), and closed heat injury (**D**) on mortality

Many included studies focused on the effects of TH on patient mortality rates. Henderson et al. [21] failed to show any reduction in mortality rates in the exposure group compared to the control group. Leng et al. [34] reported no significant difference in mortality between the intervention and control groups. Kim et al. [39] reported that TH didn’t affect the mortality rate. Graphical representation of overlap between studies in this section is provided in Fig. 2

#### Prophylactic hypothermia

Studies included in this section showed a very high overlap (CCA = 24% and CA = 49.33%). Fox et al. [17] applied prophylactic hypothermia to adult patients with blunt TBI and reported that the RR for mortality in the TH group was 0.73. They also performed analysis on the effects of short-and long-term hypothermia. Short-term hypothermia had no beneficial impact on mortality; however, long-term hypothermia showed an RR of 0.62. However, Wu et al. [29] failed to show any significant reduction in mortality with both conditions. Georgiou et al. [19] revealed an RR of 0.84 for the impact of prophylactic TH on mortality, but the results weren`t reproduced when only high-quality studies were included.

#### Adults

Overlap analysis revealed a CCA and CA of 40.1% and 48.66%, respectively, which is considered a very high overlap. Chen et al. [13] reported that the RR of TH can reduce mortality when used in early TBI or as a treatment, but the results from low-risk-of-bias studies were not in line with previous findings.

McIntyre et al. [22] reported a reduction in the mortality of patients in the TH. They also reported this effect to be present at hypothermia with a duration of >48 h. Olah et al. [36] reports a similar reduction in overall mortality and a decrease in TH for >48 h. Their subgroup analysis revealed that faster rewarming may affect mortality, as the mortality rate at < 0.25^◦^C/h was lower. Peterson et al. [26] showed similar effects in TH >48 h, but failed to show an overall reduction in mortality. Zang et al. [35] reported a reduction in mortality in the TH group based on data from 19 studies. Zhu et al. [33] and Huang et al. [38] reported no differences in mortality between the two groups.

#### Children

A very high overlap was observed between the studies included in this section (CCA = 62.5% and CA = 75%). Zhang et al. [27] reported that the RR of mortality in the TH group was 1.84, and this increase in mortality was present in the subgroup analysis of patients with GCS ≤ 8. Ma et al. [32] has a high overlap with Zhang et al. [27], and the results were expected to be similar—likewise, Geng et al. [30] reported a greater mortality rate in the TH group.

#### Closed head injury

The calculated overlap between the studies of this section resulted in a CCA of 30.49% and a CA of 40.42%, which indicates a very high overlap. A review of 23 RCTs by Sydenham et al. [24] didn`t observe any significant reduction in mortality. Alderson et al. [16] has an overlap of 81.8% with the previous study, and their conclusions are the same.

The meta-analysis by Du et al. [25] also didn`t reveal any significant reduction in mortality. Utsumi et al. [23] has an 85.7% overlap with Du et al. [25], with similar results. Crossley et al. [14] reported an increase in deaths in the normothermia group, which was present in the analysis of low-risk-of-bias RCTs.

Watson et al. [28] and Maryniuk et al. [31] also included closed-head injuries and had a CCA of 60.7%. Watson et al. [28] reported that the RR of mortality in the TH group was lower, and this protective effect was observed at the 6-month follow-up but not at the 3-month follow-up. However, this effect wasn`t observed in the analysis of studies with a low risk of bias. Martyniuk et al. [31] also reported a similar protective effect of TH on mortality, which was not present in the analysis with a low risk of bias. In a subgroup analysis based on the method of hypothermia, they reported that the protective effect of TH is only present in systemic surface cooling methods such as cooling blankets.

### Morbidity

**Fig. 3:**
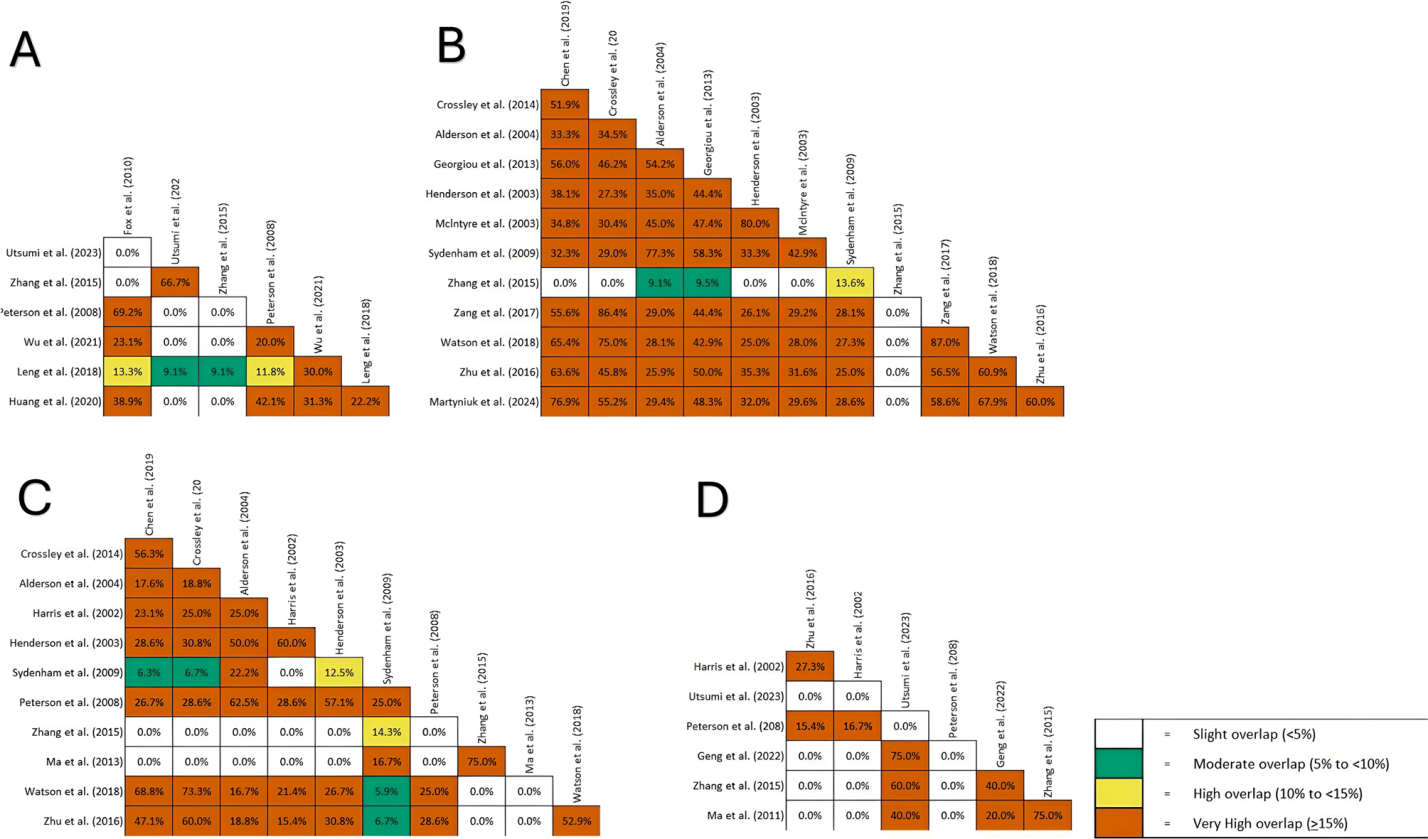
Graphical representation of the overlap between the studies investigating the effects of TH on favorable neurologic outcome (**A**), unfavorable neurologic outcome (**B**), pneumonia (**C**) and arrythmia (**D**)

Studies have investigated the morbidity of patients treated with two different approaches. First, they defined the unfavorable neurologic outcomes. Then, favorable [17, 23, 26, 27, 29, 34] or unfavorable [13, 14, 16, 19, 21, 22, 24, 27, 28, 31, 33] neurologic outcomes were measured at the end of the follow-up period. Du et al. [25] was the only study that analyzed the mean difference in GOS between the two groups. Their meta-analysis included two RCTs with 72 pediatric patients after a TBI and found a significant 1.16 mean difference. The graphical representation of overlap between studies is presented in Fig. 3.

#### Favorable neurologic outcomes

A very high overlap (CCA = 19.05% and CA = 30.61%) was observed between the studies included in this section. Fox et al. [17] reported that the RR of favorable neurological outcomes in the TH group was 1.52. Similar results were also reported in long-term hypothermia (>48 h), while short-term hypothermia didn`t increase GOS significantly. Peterson et al. [26] failed to show the effect of TH on the morbidity of patients with TBI. However, they showed that hypothermia could benefit patients who received long-term hypothermia or didn’t receive barbiturates. Huang et al. [38] reported more favorable neurological outcomes in the TH group, which was also observed in a subgroup analysis of 12-and 24-month follow-ups but was not present in the 3- and 6-month periods. The calculated overlap between each pair of studies that showed a significant GOS increase in TH showed a very high overlap between 38.9% and 69.2%.

Based on data from 5 trials and 426 patients, Utsumi et al. [23] failed to show an increase in favorable neurological outcomes. Zhang et al. [27] calculated the RRs of the GOS 4–5 score in the TH group at 3 and 6-month follow-ups to be 0.89 and 0.91, respectively. The CCA between both these studies was calculated to be 66.7%. This high overlap may be due to the selection of a similar pediatric population in both studies. Wu et al. [29] investigated the effect of prophylactic hypothermia on the morbidity of TBI patients. Prophylactic hypothermia didn’t affect the neurological outcome of TBI patients. Leng et al. [34] reported no significant differences in favorable neurological outcomes between the two groups.

#### Poor neurological outcomes

Analysis revealed a very high overlap between the studies of this section, with a CCA of 35.06% and CA of 40.48%. Zhang et al. [27] had the lowest overlap with other systematic reviews in this section, and its meta-analysis revealed no significant decrease in unfavorable neurological outcomes in TH. Similarly, Alderson et al. [16], Henderson et al. [21], & Zhu et al. [33] also failed to show a significant reduction in poor neurological outcomes.

On the contrary, Mclntyre et al. [22] reported that the RR of poor neurological outcomes was 0.78, and this benefit was present in patients with a hypothermia duration of 24 h or ≥ 48 h, but not in those with a duration of 48 h. Similarly, Zang et al. [35], Chen et al. [13], & Crossley et al. [14] reported that hypothermia decreased poor outcomes in patients. After removing studies with a high risk of bias, the effect of TH on poor neurological outcomes was still present.

Unlike previous paragraphs, most systematic reviews report fewer unfavorable neurological outcomes in TH; however, these beneficial effects disappear after excluding low-quality studies. Georgiou et al. [19] proposed that prophylactic hypothermia can reduce poor neurological outcomes. However, in the subgroup analysis of high-quality studies, this effect of prophylactic hypothermia was not detected. Sydenham et al. [24] reported that the OR of unfavorable outcomes was 0.77. Nevertheless, the analysis of high-quality studies alone didn’t show any protective effects of hypothermia. Watson et al. [28] followed adults with closed-head injuries and reported that TH had a protective effect, but the effect disappeared in the analysis of low-risk-of-bias studies. Martyniuk et al. [31] reported a similar disappearance of protective effects of hypothermia in low-risk-of-bias studies. They also state that the method of hypothermia can affect the outcome, and systemic cooling or isolated cranial cooling methods had the greatest effects on the outcome of TBI.

### Infection and pneumonia

One study in the review by Galvin et al. [18], reported RR of 1.77 for surgical site infection in the hypothermia group, who also underwent brain surgery. In contrast, both Geng et al. [30] and Geurts et al. [40] reported revealed no difference in the infection rate between the TH and normothermia groups.

The graphical representation of studies in this section is provided in Fig. 3. The analysis revealed a CCA and CA of 21.92% and 29.02% which is considered a very high overlap. The study by Fox et al. [17] reported no difference in the incidence of pneumonia between the two groups, but the authors did not provide the data needed for the analysis. Eleven reviews conducted meta-analyses of pneumonia rates in the TH group [13, 14, 16, 20, 21, 24, 26–28, 32, 33].

Sydenham et al. [24] reported a nonsignificant increase in pneumonia in the TH group—likewise, Crossley et al. [14], Harris et al. [20], and Henderson et al. [21] failed to show increased rates of pneumonia in TH in their meta-analysis. In similar studies by Zhang et al. [27] and Ma et al. [32], the pneumonia risk between the two groups was the same. Studies that didn`t show a difference in pneumonia showed heterogeneous overlap results (between 0% and 75%).

However, a more recent meta-analysis by Chen et al. [13] reported a significantly greater risk for pneumonia in the TH group (1.48). Similarly, Alderson et al. [16] reported that the OR of pneumonia in the TH group was 1.95. Peterson et al. [26] reported an increase in the incidence of pneumonia in the TH group, and the risk further increased when the TH group also took barbiturates. Two studies by Watson et al. [28] and Zhu et al. [33], reported that the rate of pneumonia increased in the TH group. When considering the overlap between two studies, all those reports of high pneumonia rate in the TH group have a high CCA (between 16.7% and 68.8%). This high overlap may explain the similarity between the results.

### Other complications

Two studies investigated the incidence of complications in the TH group compared to the control group. Both Du et al. [25] and Geng et al. [30] reported no difference in adverse outcomes between the two groups.

#### Arrhythmia

The reported overlap for this section, with a CCA of 12.96% and CA of 25.4% was high. Figure 3 provides a graphical representation of the overlap of studies in this section. In a study by Zhu et al. [33], cardiac complications were shown to be higher in the TH group. In six studies [20, 23, 26, 27, 30, 32], arrhythmia was assessed in a 30–35 °C range. Harris et al. [20] reported that hypothermia is not associated with arrhythmia. Utsumi et al. [23] failed to observe a lower incidence of arrhythmia in the normothermia group compared to patients under different hypothermia durations. They also performed a network meta-analysis and reported no difference between the duration of hypothermia and arrhythmia (p-score of 72 h hypothermia = 0.29, p-score of 48 h hypothermia = 0.36, p-score of 24 h hypothermia = 0.63). Neither of the studies by Peterson et al. [26] and Geng et al. [30] reported a significant difference in the incidence of arrhythmia. However, Zhang et al. [27] and Ma et al. [32] both reported a higher risk of arrhythmia in children receiving TH.

#### Coagulopathy and bleeding

Harris et al. [20] reported that TH at a target temperature of 32–33 °C didn`t affect the prothrombin time but may increase the partial thromboplastin time (OR = 2.22). Zhang et al. [27] and Zhu et al. [33] both reported no increase in the risk of bleeding associated with hypothermia.

#### Electrolyte abnormalities

Fox et al. [17] reported hypokalemia as the most common electrolyte abnormality in 6 RCTs. The authors did not provide data about the rate of hypokalemia but stated that it was treated without any sequelae.

## Discussion

The included studies showed controversial results regarding the effects of TH on the mortality and morbidity of TBI patients. This may be due to methodological differences and the quality of evidence that studies provide. Trials investigating the results of TH on TBI differ in many ways. The target temperature, differences between prophylactic TH and treatment TH, induction and duration of TH, method of TH delivery, type of TBI injuries, target ICP, rewarming rate, GCS score at admission, and age of TBI patients are all factors that need to be considered in this field. Almost all studies included in this review applied TH interventions for > 12 h. There are no clear guidelines about the duration of TH in TBI patients, and some studies have targeted the ICP to define the time to the end of TH.

We didn’t include studies by Signorini et al. [42], Christian et al. [43], Saxena et al. [44], Li et al. [45], Crompton et al. [46], Sadaka et al. [47], Madden et al. [48], Naseri Alavi et al. [49], Florez et al. [50], or Keeves et al. [51] since they didn’t fulfill our inclusion criteria. Our work also differed from a previous umbrella review by Moore et al. [52] since we investigated a narrower research question with more details, including more updated systematic reviews, and didn’t limit our target population to only adults.

Although theoretically, TH may improve the outcome of TBI patients [53, 54], the result of the included studies vary regarding the benefits of TH. There is a shift in the beneficial effect of TH on mortality and the neurological outcome of TBI patients. Especially in the more recent systematic reviews, which have a higher quality and include recent high-quality RCTs such as the Eurotherm3235 [55] and the POLAR-RCT [56]. TH failed to improve the mortality and morbidity of TBI patients. This conclusion was further supported when the meta-analysis was conducted exclusively in low-risk-of-bias or high-quality studies.

The main adverse effects of TH investigated in studies were pneumonia, arrhythmia, and bleeding. Most of the included studies showed an increased risk of pneumonia in the TH group. Except for Sydenham et al. [24], studies reporting no difference in pneumonia rates had fewer than 10 RCTs. This may explain their results and the low CCA. Mechanical ventilation, prolonged hospital stays, and lower consciousness are risk factors for pneumonia in TBI patients [57, 58]. Also, the diagnosis of pneumonia in TBI patients is challenging since fever and leukocytosis are common in them, and TH reduces the leukocyte count and C-reactive protein levels [59–61].

The risk of arrhythmia in TH patients in the included studies was controversial. This may be due to differences in the sample size or methodology of the studies. However, pediatric patients seemed to be at a greater risk of arrhythmia in the TH group. Traumatic patients are at risk of arrhythmia because of different etiologies (e.g., trauma to the chest, electrolyte abnormalities, underlying cardiac disease). A temperature of 32–34 °C is reported not to cause life-threatening arrhythmias [62]. while the temperature ≤ 30^◦^C significantly increases the risk of arrhythmia [63, 64].

The studies included in our review did not show a significant risk of bleeding at 32–35 °C. Studies in TBI patients have demonstrated that 32–34 °C doesn`t significantly increase coagulopathy [65]. Another study reported ≥ 33^◦^C to be a safe temperature [64]. The application of TH after craniotomy in patients with TBI reportedly results in favorable neurologic outcomes during a 12-month follow-up period [66]. It seems that the best time to apply TH in TBI patients with the lowest hemorrhage risk is after controlling active sources of bleeding or craniotomy.

## Conclusion

This umbrella review provides an overview of the effectiveness of TH in managing TBI patients. According to the Advanced Trauma Life Support (ATLS) guidelines, when traumatic patients are admitted to the emergency department, their management should be focused on events that are life-threatening for the patients (e.g., controlling visible external bleeding, having a secure airway) [67]. TBI can be a life-threatening event, and hypothermia is a proposed treatment and prophylaxis for lowering the ICP. However, there are multiple differences and controversies between the methods and results of studies. For example, the results of meta-analysis change when the subgroup analysis of high-quality studies is performed or when the target temperature and duration of TH differ between studies. Also, newer high-quality RCTs and systematic reviews fail to show the proper impact of TH on mortality and morbidity. These controversies promote the need for more high-quality studies with specific protocols to determine the possible use of TH in TBI.

## Limitations

This study has potential limitations. Our review only included articles written in English; however, there are articles focusing on the same issue but published in other languages, such as Spanish. We also did not search databases such as LILACS and SciELO, which provide a useful resource for non-English articles. This language barrier may affect the generalizability of our study’s conclusions, as it decreases the heterogeneity of races included. One major limitation of umbrella reviews is the difference between the methodologies of included studies. Studies differed in the method, target temperature, duration of TH, and the rewarming rate of patients. A direct comparison of these parameters was impossible, and we couldn’t perform any analysis. Pediatric patients may also show a different response to TH than adults. Also, there are more advanced formulas for overlap analysis, such as sCCA and wCCA [68, 69], but since there are currently no tools such as GROOVE for these formulas, we were unable to use them. For future studies, we suggest using TH in multiorgan damage TBI patients and comparing various methods for delivering TH. Studies could also investigate different targets for the duration of TH and rewarming rates to determine the best settings for each patient.

## Supplementary Information

Supplementary Material 1.

Supplementary Material 2.

Supplementary Material 3.

## Data Availability

The authors confirm that the data supporting the findings of this study are available within the article and its supplementary materials.

## Abbreviations

ATLS: Advanced Trauma Life Support
CI: Confidence Interval
GCS: Glasgow Coma Scale
GOS: Glasgow Outcome Scale
ICP: Intracranial Pressure
JBI: Joanna Briggs Institute
OR: Odds Ratio
RCT: Randomized Controlled Trial
RR: Relative Risk
TBI: Traumatic Brain Injury
TH: Therapeutic Hypothermia
