# Supplementary material for: Therapeutic hypothermia in patients with traumatic brain injury: an umbrella review
Source: BMC Neurol. 2025 Oct 24;25:440. doi: 10.1186/s12883-025-04463-3 (PMC12553179; doi:10.1186/s12883-025-04463-3)
Supplement: Supplementary file 1 — Supplementary Material 1. [file 12883_2025_4463_MOESM1_ESM.pdf]

Search query and search strategies:

PubMed

(((((((TBI[Text Word]) OR (TBIs[Text Word])) OR ((((((((((Craniocerebral[Text Word]) OR (Brain[Text Word])) OR (Skull[Text Word])) OR (Head[Text Word])) OR (Encephalopath*[Text Word])) OR (Parietal[Text Word])) OR (Occipital[Text Word])) OR (Forehead[Text Word])) OR (Frontal[Text Word])) AND ((Trauma*[Text Word]) OR (injur*[Text Word])))) OR ("Brain Injuries, Traumatic"[Mesh])) OR ("Craniocerebral Trauma"[Mesh:NoExp])) AND (("Hypothermia"[Mesh]) OR (Hypothermia*[Text Word]))) AND (("Systematic Review" [Publication Type]) OR "Meta-Analysis" [Publication Type])) OR ((((((TBI[Text Word]) OR (TBIs[Text Word])) OR ((((((((((Craniocerebral[Text Word]) OR (Brain[Text Word])) OR (Skull[Text Word])) OR (Head[Text Word])) OR (Encephalopath*[Text Word])) OR (Parietal[Text Word])) OR (Occipital[Text Word])) OR (Forehead[Text Word])) OR (Frontal[Text Word])) AND ((Trauma*[Text Word]) OR (injur*[Text Word])))) OR ("Brain Injuries, Traumatic"[Mesh])) OR ("Craniocerebral Trauma"[Mesh:NoExp])) AND (("Hypothermia"[Mesh]) OR (Hypothermia*[Text Word])) AND (meta-analysis[Filter] OR systematicreview[Filter]))

CINAHL:

#	Query	Limiters/Expanders	Last Run Via
S15	S13 OR S14	Expanders - Apply equivalent subjects<br>Search modes - Find all my search terms	Interface - EBSCOhost Research Databases<br>Search Screen - Advanced Search<br>Database - CINAHL with Full Text
S14	S5 AND S8	Limiters - Publication Type: Meta Analysis, Systematic Review<br>Expanders - Apply	Interface - EBSCOhost Research Databases

		equivalent subjects<br>Search modes - Find all my search terms	Search Screen - Advanced Search<br>Database - CINAHL with Full Text
S13	S11 AND S12	Expanders - Apply equivalent subjects<br>Search modes - Find all my search terms	Interface - EBSCOhost Research Databases<br>Search Screen - Advanced Search<br>Database - CINAHL with Full Text
S12	S5 AND S8	Expanders - Apply equivalent subjects<br>Search modes - Find all my search terms	Interface - EBSCOhost Research Databases<br>Search Screen - Advanced Search<br>Database - CINAHL with Full Text
S11	S9 OR S10	Expanders - Apply equivalent subjects<br>Search modes - Find all my search terms	Interface - EBSCOhost Research Databases<br>Search Screen - Advanced Search<br>Database - CINAHL with Full Text
S10	(MH "Meta Analysis")	Expanders - Apply equivalent subjects<br>Search modes - Find all my search terms	Interface - EBSCOhost Research Databases<br>Search Screen - Advanced Search<br>Database -

			CINAHL with Full Text
S9	(MH "Systematic Review")	Expanders - Apply equivalent subjects<br>Search modes - Find all my search terms	Interface - EBSCOhost Research Databases<br>Search Screen - Advanced Search<br>Database - CINAHL with Full Text
S8	S6 OR S7	Expanders - Apply equivalent subjects<br>Search modes - Find all my search terms	Interface - EBSCOhost Research Databases<br>Search Screen - Advanced Search<br>Database - CINAHL with Full Text
S7	(MH "Hypothermia")	Expanders - Apply equivalent subjects<br>Search modes - Find all my search terms	Interface - EBSCOhost Research Databases<br>Search Screen - Advanced Search<br>Database - CINAHL with Full Text
S6	Hypothermia*	Expanders - Apply equivalent subjects<br>Search modes - Find all my search terms	Interface - EBSCOhost Research Databases<br>Search Screen - Advanced Search<br>Database - CINAHL with Full Text

S5	S1 OR S2 OR S3 OR S4	Expanders - Apply equivalent subjects<br>Search modes - Find all my search terms	Interface - EBSCOhost Research Databases<br>Search Screen - Advanced Search<br>Database - CINAHL with Full Text
S4	(TBI OR TBIs)	Expanders - Apply equivalent subjects<br>Search modes - Find all my search terms	Interface - EBSCOhost Research Databases<br>Search Screen - Advanced Search<br>Database - CINAHL with Full Text
S3	((Craniocerebral OR Brain OR Skull OR Head OR Encephalopath* OR Parietal OR Occipital OR Forehead OR Frontal) N5 (Trauma* OR injur*))	Expanders - Apply equivalent subjects<br>Search modes - Find all my search terms	Interface - EBSCOhost Research Databases<br>Search Screen - Advanced Search<br>Database - CINAHL with Full Text
S2	(MH "Brain Injuries+")	Expanders - Apply equivalent subjects<br>Search modes - Find all my search terms	Interface - EBSCOhost Research Databases<br>Search Screen - Advanced Search<br>Database - CINAHL with Full Text
S1	(MH "Head Injuries")	Expanders - Apply equivalent subjects	Interface - EBSCOhost Research

		Search modes - Find all my search terms	Databases Search Screen - Advanced Search Database - CINAHL with Full Text

Cochrane:

ID	Search
#1	MeSH descriptor: [Craniocerebral Trauma] this term only
#2	MeSH descriptor: [Brain Injuries, Traumatic] explode all trees
#3	((Craniocerebral OR Brain OR Skull OR Head OR Encephalopath* OR Parietal OR Occipital OR Forehead OR Frontal) NEAR/5 (Trauma* OR injur*)):ti,ab,kw (Word variations have been searched)
#4	(TBI OR TBIs):ti,ab,kw (Word variations have been searched)
#5	#1 OR #2 OR #3 OR #4
#6	MeSH descriptor: [Hypothermia] explode all trees
#7	(Hypothermia*):ti,ab,kw (Word variations have been searched)
#8	#6 OR #7
#9	#5 AND #8

Embase:

No.	Query
#13	#10 OR #12
#12	#9 AND #11
#11	'systematic review'/exp OR 'meta analysis'/exp
#10	#5 AND #8 AND ([cochrane review]/lim OR [systematic review]/lim OR [meta analysis]/lim)
#9	#5 AND #8
#8	#6 OR #7
#7	hypothermia*:ab,ti,kw
#6	'hypothermia'/exp
#5	#1 OR #2 OR #3 OR #4
#4	tbi:ab,ti,kw OR tbis:ab,ti,kw
#3	((craniocerebral OR brain OR skull OR head OR encephalopath* OR parietal OR occipital OR forehead OR frontal) NEAR/5 (trauma* OR injur*)):ab,ti,kw
#2	'traumatic brain injury'/exp
#1	'head injury'/de

Scopus:

((TITLE-ABS-KEY (craniocerebral OR brain OR skull OR head OR encephalopath* OR parietal OR occipital OR forehead OR frontal) W/5 TITLE-ABS-KEY (trauma* OR injur*)) OR (TITLE-ABS-KEY (tbi OR tbis))) AND (TITLE-ABS-KEY (hypothermia*)) AND (TITLE-ABS-KEY (systematic W/3 review*) OR TITLE-ABS-KEY (meta-analysis) OR TITLE-ABS-KEY ("Meta Analysis"))

Web of Science:

7. #5 AND #6
6. Systematic NEAR/3 Review* (Topic) or Meta-Analysis (Topic) or "Meta Analysis" (Topic)
5. #4 AND #3
4. #1 OR #2
3. Hypothermia* (Topic)
2. (TBI OR TBIs) (Topic)
1. (Craniocerebral OR Brain OR Skull OR Head OR Encephalopath* OR Parietal OR Occipital OR Forehead OR Frontal) NEAR/5 (Trauma* OR injur*) (Topic)

Epistemonikos:

(title:((title:(TBI OR TBIs) OR abstract:(TBI OR TBIs)) AND (title:(Hypothermia*) OR abstract:(Hypothermia*))) OR abstract:((title:(TBI OR TBIs) OR abstract:(TBI OR TBIs)) AND (title:(Hypothermia*) OR abstract:(Hypothermia*)))) OR (title:((title:(Craniocerebral OR Brain OR Skull OR Head OR Encephalopath* OR Parietal OR Occipital OR Forehead OR Frontal) OR abstract:(Craniocerebral OR Brain OR Skull OR Head OR Encephalopath* OR Parietal OR Occipital OR Forehead OR Frontal)) AND (title:(Trauma* OR injur*) OR abstract:(Trauma* OR injur*)) AND (title:(Hypothermia*) OR abstract:(Hypothermia*))) OR abstract:((title:(Craniocerebral OR Brain OR Skull OR Head OR Encephalopath* OR Parietal OR Occipital OR Forehead OR Frontal) OR abstract:(Craniocerebral OR Brain OR Skull OR Head OR Encephalopath* OR Parietal OR Occipital OR Forehead OR Frontal)) AND (title:(Trauma* OR injur*) OR abstract:(Trauma* OR injur*)) AND (title:(Hypothermia*) OR abstract:(Hypothermia*))))
