# Supplementary material for: Therapeutic hypothermia in patients with traumatic brain injury: an umbrella review
Source: BMC Neurol. 2025 Oct 24;25:440. doi: 10.1186/s12883-025-04463-3 (PMC12553179; doi:10.1186/s12883-025-04463-3)
Supplement: Supplementary file 2 — Supplementary Material 2. [file 12883_2025_4463_MOESM2_ESM.pdf]

A. Important parameters in the therapeutic hypothermia intervention based on the PICO process

**Population**	**Intervention**	**Comparison**	**Outcome**
• Adult or pediatrics<br>• Number of participants<br>• Closed TBI<br>• GCS of patients at admission<br>• ICP at the admission	• Hypothermia for ICP prophylaxis of high ICP treatment<br>• interval between trauma hypothermia<br>• Cooling rate<br>• Duration of hypothermia<br>• Target temperature<br>• Target ICP<br>• Method of cooling<br>• Use of barbiturate<br>• Rewarming rate<br>• Need for neurosurgical intervention	• Did the normothermia group have any intervention to keep the body temperature at a target temperature, or was the temperature controlled passively<br>• Control temperature?	• Mortality<br>• Unfavorable neurologic outcome<br>• Favorable neurologic outcome<br>• Pneumonia<br>• Bleeding and coagulopathy<br>• Arrhythmia<br>• Electrolyte abnormalities

ICP: Intracranial pressure, TBI: traumatic brain injuries

B. Characteristics of included studies

Author/published year	Country	Bias risk assessment and quality checklist	Number and type of included studies	Number of participants (intervention group/control group)	Pediatrics or adults?	GCS of included studies	Type of TBI	Induction time of hypothermia	Duration of hypothermia	Methods to apply hypothermia	Target temperature (°C)	The fastest rewarming rate	Unfavorable neurologic outcome	Follow up length
Harris et al. (2002) (1)	USA	"Assessing the quality of reports of randomized clinical trials: is blinding necessary?" by Jadad et al. (2)	7 RCT	668	-	-	-	6 h – mean of 15 h post-injury	≤ 14 days (mostly 48 h)	-	32 - 35	From 12h – 5 days	-	-
Henderson et al. (2003) (3)	Canada	assessed studies based on the blinded assessment of the outcome an explanation of their randomization method	8 RCT	748	-	≤ 9	-	< 6 h	1-14 days	-	32 - 35	-	GOS ≤ 3	6 months
Mclntyre et al. (2003) (4)	Canada	allocation concealment and blinding of the outcome assessment were used to assess the methodology	12 RCT	1069 (543/526)	Adults	Mean GCS < 8	-	-	1-14 days	-	30 – 34.5	≤ 1°C/h	GOS ≤ 3	3,6,12 months
Alderson et al. (2004) (5)	UK	authors used adequacy of allocation concealment and the degree of blinding in the assessment of outcome instead of any checklists	14 RCT	1094	Pediatrics and adults	≤ 8	Closed head injury	Within 10 hours after trauma	1-4 days	Cooling blanket, ice, cold gastric lavage, unwarmed ventilator gases, temperature control pads,	30 – 34.5	≤ 0.5°C/2h	GOS ≤ 3	3,6,12 months

Peterson et al. (2008) (6)	USA	The quality assessment tool was created based on criteria developed by the U.S. Preventive Services Task Force (Harris et al., 2001), the National Health Service Centre for Reviews and Dissemination (U.K.) (Centre for Reviews and Dissemination, 2001), and the Cochrane Collaboration (Higgins and Green, 2006).	13 RCTs	1339 (689/650)	adults	Mean GCS <6	-	Immediately-20h	1-14 days	-	30-35	≤ 1˚C/h	GOS ≤ 3	120 h, 3, 6, 12, 24 months
Sydenham et al. (2009) (7)	UK	The Cochrane Collaboration 2008; Vol. Version 5.0.0 [updated February 2008]	23 RCT	1614	Pediatrics and adults	≤ 9	closed head injury	6-48h	1-8 days	Cooling blankets, Cooling bed, Ice blocks, iced saline stomach lavage	30-35	≤ 1˚C/3h	GOS ≤ 3	1, 3, 6, 12 months
Fox et al. (2010) (8)	Canada	Blinding and Allocation concealment	12 RCT	1327 (670/657)	Adult	≤ 8	Blunt TBI	-	1-14 days: Short (<48h) long (>48)	-	32-35	-	GOS ≤ 3	-
Harris et al. (2012) (9)	UK	Randomized controlled trials were assessed for adequacy of the randomization and allocation	1 RCT	25 (12/13)	Adult	≤ 8	-	≤ 48	24 h	Head and neck cooling	33	≤ 0.5˚C/3h	-	1 month

		concealment process, the potential for selection bias after allocation, and level of masking												
Georgiou et al. (2013) (10)	UK	GRADE	18 RCT	1851	Pediatrics and adults	≤ 8 (only high and moderate-quality studies were reported)	-	-	24-103h	-	30 - 35	≤ 1˚C/h	GOS ≤ 3 in adults PCPC 3-6 in children	3, 6, 12 months
Ma et al. (2013) (11)	China	Random allocation and baseline demographic similarity were used for quality assessment	6 RCTs	366	Pediatrics	Mean GCS <8	-	-	1 to >3 days	-	32-34.7	≤ 1˚C/h	-	-
Crossley et al. (2014) (12)	UK	Cochrane Collaboration tool for assessing risk of bias	20 RCT	1885 (999/886)	Adult	-	Closed head injury	2.5 – 20 h	Average of 4.5 h on 3 consecutive days to 14 d	Cooling blankets, Application of ice or gastric lavage with ice, cold saline gastric lavage, IV cold crystalloid, gel packs, air cooling, circulating water mattress, Ice pillow, Cooling cap	< 36	≤ 0.3˚C/h	GOS ≤ 3 or equivalent scoring in Ranchos Los Amigos scale	3-6 months
Geurts et al. (2014) (13)	Netherlands	Cochrane Risk of Bias Methods (14)	10 clinical trials (out of 23 trials)	524 (264/260)	Adults	-	-	-	1-14 days	Surface cooling	33-35.5	-	-	3, 6, 12, 24 months
Galvin et al. (2015) (15)	Canada	Cochrane Collaboration tool for	1 (out of 4) RCT	80 (40/40)	Adults	≤ 8	non-penetrating severe TBI	Immediately after the operation	4 days	Water-circulating cooling	33 - 35	-	GOS ≤ 3	12 months

		assessing risk of bias								blanket, Cooling cap, refrigerated ice bags				
Zhang et al. (2015) (16)	China	Cochrane Reviewer's Handbook 5.1.0	7 RCTs	442 (218/224)	Pediatrics	≤ 15 (9 patients with GCS >8 and the rest had GCS ≤8)	-	Less than 8 hours after trauma	1-3 days	Saline, cooling blankets, ice cap	32-34	≤ 1°C/4h	GOS ≤ 3	3, 6, 12 months
Zhu et al. (2016) (17)	China	modified Jadad score, Cochrane Handbook for Systematic Reviews of Interventions	18 RCTs	2177 (1122/1055)	Adults	-	-	Within 24h	24-366 h	-	32-35	≤ 1°C/h	GOS ≤ 3	3 and 6 months
Dunkley e. al. (2017) (18)	UK	CASP randomized controlled trial checklist or the CASP cohort study checklist	8 (6 RCTs/ 2 retrospective quasi-experimental studies)	689 (493/196)	Adult	≤ 8	-	-	2-5 days	-	33-35	≤ 1°C/h	GOS ≤ 3	6, 12, 24 months
Lewis et al. (2017) (19)	UK	Cochrane Risk of Bias Tool and GRADE	37 RCTs	3110 (1556/1554)	Pediatrics and adults	≤ 8	Both open and close	3 hours after injury – 10 days (Some studies reported immediately after admission)	1-14 days (until ICP normalizes)	Cooling blanket, Cold IV saline, Crushed ice exposed to the surface, iced saline gastric lavage, cooling cap, cold irrigation of Hartmann's solution onto the surface of the brain, forced air, ice bags	30 - 35	≤ 1°C/h	GOS ≤ 3	1, 3, 6, 12 months

Zang et al. (2017) (20)	China	Cochrane Collaboration's tool for evaluating the risk of bias (14)	21 RCTs	2275 (1199/1076)	Adult	-	-	-	1-7 days	IV refrigerated saline, cooling blanket, wet sheet, gel packs, cold gastric lavage, ice pillow, room temperature air in ventilator cooling	32-35	-	GOS ≤ 3 or equivalent scoring scale	3, 6, 12, 24 months
Leng et al. (2018) (21)	China	Quality assessment was done based on the similarity between baseline characteristics of groups and random allocation	7 clinical trials	1331 (689/642)	-	≤ 8	-	3-10h	24-48 h	-	32-36	The fastest rewarming time was 4h	GOS ≤ 3	3, 6, and 12 months
Olah et al. (2018) (22)	Hungary	Cochrane Handbook (14), The Jadad score (2), level of randomization	14 RCTs	1786 (896/890)	adults	≤ 8	-	-	24-336h	Whole body cooling	32-36	≤ 1˚C/h	-	-
Watson et al. (2018) (23)	UK	Cochrane Collaboration's tool for assessing the risk of bias	22 RCTs	2346 (1240/1106)	Adults	≤ 8	closed head injury	-	-	-	<36	-	-	3 to >6 months
Chen et al. (2019) (24)	China	The Cochrane Collaboration's tool and the Jadad score	23 RCT	2796 (1448/1348)	Adults	≤ 15	-	Immediately – 10 days: Early TH (<24h) Late TH (>24h)	1-14 days	-	30 - 36	≤ 1˚C/h	GOS ≤ 3 or GOS-E ≤ 4	3, 6, 12, 24, 48 months
Meizikri et al. (2019) (25)	Indonesia	GRADE	2 RCTs	127 (63/64)	Children and adolescent	Median ≤6	-	< 6h after injury	-	-	32-33	< 1˚C/3h	-	3, 6, and 12 months

Huang et al. (2020) (26)	China	The Cochrane Risk Assessment Tool (14)	15 RCTs	2523 (1320/1203)	Adults	≤ 8	-	Immediately – 10 days	1-14 days	-	32-35	≤ 1°C/h	GOS ≤ 3 or GOS-E ≤ 4	3, 6, 12, and 24 months
Kim et al. (2020) (27)	Italy	Cochrane Handbook for Systematic Reviews of Interventions	9 (out of 14 randomized trials)	1900 (986/914)	Pediatrics and adults	-	-	-	Minimum of 24-72h	Cold saline infusion	32-35	-	GOS ≤ 3, GOS-E ≤ 4, PCPC ≥ 4, and PE-GOS ≥ 5	3,6, 12 months
Wu et al. (2021) (28)	China	Cochrane Collaboration Risk of Bias Tool	6 RCTs	1225 (657/568)	Adults	≤ 8	-	Within 6h after the injury	1-7 days	Cooling blanket, nasogastric lavage with iced fluids, IV cooling, room temperature air in the ventilator circuit	32-35	≤ 1◦C/h	GOS ≤ 3 GOS-E ≤ 4	6 months
Du et al. (2022) (29)	China	Cochrane Handbook of Systematic Evaluation of Interventions (30)	8 RCTs	514 (257/257)	Pediatrics	≤ 8	-	8h After Trauma	1-7 days	Cooling blanket, cooling cap	32-35	-	-	-
Geng et al. (2022) (31)	China	Cochrane Review Handbook 5.3	5 RCTs	421 (206/215)	Pediatrics	-	-	-	-	-	-	-	-	
Utsumi et al. (2023) (32)	Japan	Cochrane Collaboration's RCT Risk of bias Tool	6 RCT	448 (220/228)	Pediatrics	< 8	closed head injury	-	1-3 days	-	32 - 34.5	-	CPC ≥ 3, GOS ≤ 3, Pediatric CPC ≥ 4, pediatric version of the GOS-E ≤ 4	3 months or more
Martyniuk et al. (2024) (33)	Canada	revised Cochrane risk of bias tool for randomized trials (RoB 2), GRADE	32 RCTs	3909 (2105/1894)	Adults	≤ 12	Blunt TBI	-	1-14 days	Cooling cap, cooling neck blanket, water circulating blanket, gastric	32-35	-	GOS ≤ 3 GOS-E ≤ 4	1, 3, 6, 12, 24 months-7 years

										lavage, hypothermia machine with cooling blankets, medical cooling bed, ice bag, chilled fluid irrigation along the subdural catheter, rapid cold infusion, room temperature air in ventilator, gel pads				

CASP: Critical Appraisal Skills Program, CPC: Cerebral Performance Category, GCS: Glasgow coma scale, GOS: Glasgow outcome scale, GOS-E: Glasgow outcome scale-extended, GRADE: Grading of Recommendations Assessment, Development, and Evaluation, IV: intravenous, PCPC = Pediatric Cerebral Performance, PE-GOS = Pediatric Extended Glasgow Outcome Scale, PRISMA: The preferred reporting items for systematic reviews and meta-analyses statement, PICU: pediatric intensive care unit, QUOROM: quality of reporting of meta-analyses statement, RCT: Randomized controlled trial, TBI: Traumatic brain injury, TH: therapeutic hypothermia,

1. Harris, O.A., et al., *The role of hypothermia in the management of severe brain injury: a meta-analysis.* Arch Neurol, 2002. **59**(7): p. 1077-83.
2. Jadad, A.R., et al., *Assessing the quality of reports of randomized clinical trials: is blinding necessary?* Control Clin Trials, 1996. **17**(1): p. 1-12.
3. Henderson, W.R., et al., *Hypothermia in the management of traumatic brain injury. A systematic review and meta-analysis.* Intensive Care Med, 2003. **29**(10): p. 1637-44.
4. McIntyre, L.A., et al., *Prolonged therapeutic hypothermia after traumatic brain injury in adults: a systematic review.* Jama, 2003. **289**(22): p. 2992-9.
5. Alderson, P., C. Gadkary, and D.F. Signorini, *Therapeutic hypothermia for head injury.* Cochrane Database Syst Rev, 2004(4): p. Cd001048.
6. Peterson, K., S. Carson, and N. Carney, *Hypothermia treatment for traumatic brain injury: a systematic review and meta-analysis.* J Neurotrauma, 2008. **25**(1): p. 62-71.
7. Sydenham, E., I. Roberts, and P. Alderson, *Hypothermia for traumatic head injury.* Cochrane Database Syst Rev, 2009(2): p. Cd001048.
8. Fox, J.L., et al., *Prophylactic hypothermia for traumatic brain injury: a quantitative systematic review.* Cjem, 2010. **12**(4): p. 355-64.
9. Harris, B., et al., *Systematic review of head cooling in adults after traumatic brain injury and stroke.* Health Technol Assess, 2012. **16**(45): p. 1-175.

10. Georgiou, A.P. and A.R. Manara, *Role of therapeutic hypothermia in improving outcome after traumatic brain injury: a systematic review.* Br J Anaesth, 2013. **110**(3): p. 357-67.
11. Ma, C., et al., *Is therapeutic hypothermia beneficial for pediatric patients with traumatic brain injury? A meta-analysis.* Child's Nervous System, 2013. **29**(6): p. 979-984.
12. Crossley, S., et al., *A systematic review of therapeutic hypothermia for adult patients following traumatic brain injury.* Crit Care, 2014. **18**(2): p. R75.
13. Geurts, M., et al., *Therapeutic hypothermia and the risk of infection: a systematic review and meta-analysis.* Critical care medicine, 2014. **42**(2): p. 231-242.
14. Higgins, J.P.T., et al., *The Cochrane Collaboration's tool for assessing risk of bias in randomised trials.* BMJ, 2011. **343**: p. d5928.
15. Galvin, I.M., et al., *Cooling for cerebral protection during brain surgery.* Cochrane Database Syst Rev, 2015. **1**(1): p. Cd006638.
16. Zhang, B.F., et al., *Meta-analysis of the efficacy and safety of therapeutic hypothermia in children with acute traumatic brain injury.* World Neurosurg, 2015. **83**(4): p. 567-73.
17. Zhu, Y., et al., *Therapeutic hypothermia versus normothermia in adult patients with traumatic brain injury: a meta-analysis.* SpringerPlus, 2016. **5**(1).
18. Dunkley, S. and A. McLeod, *Therapeutic hypothermia in patients following traumatic brain injury: a systematic review.* Nurs Crit Care, 2017. **22**(3): p. 150-160.
19. Lewis, S.R., et al., *Hypothermia for traumatic brain injury.* Cochrane Database Syst Rev, 2017. **9**(9): p. Cd001048.
20. Zang, Z., X. Xu, and S. Xu, *The efficacy of therapeutic hypothermia in adult patients with traumatic brain injury: a systematic review and meta-analysis.* Int J Clin Exp Med, 2017. **10**(6): p. 8691-9.
21. Leng, L., *Hypothermia therapy after traumatic brain injury: A systematic review and meta-analysis.* Turkish Neurosurgery, 2018. **28**(5): p. 710-715.
22. Olah, E., et al., *Therapeutic Whole-Body Hypothermia Reduces Death in Severe Traumatic Brain Injury if the Cooling Index Is Sufficiently High: Meta-Analyses of the Effect of Single Cooling Parameters and Their Integrated Measure.* J Neurotrauma, 2018. **35**(20): p. 2407-2417.
23. Watson, H.I., et al., *Revisited: A Systematic Review of Therapeutic Hypothermia for Adult Patients Following Traumatic Brain Injury.* Crit Care Med, 2018. **46**(6): p. 972-979.
24. Chen, H., et al., *A meta-analysis of the effects of therapeutic hypothermia in adult patients with traumatic brain injury.* Crit Care, 2019. **23**(1): p. 396.
25. Meizikri, R. and G. Indiradini, *Induced hypothermia for traumatic brain injury: A systematic review.* Indonesian Journal of Neurosurgery, 2019. **2**(1).
26. Huang, H.P., W.J. Zhao, and J. Pu, *Effect of mild hypothermia on prognosis of patients with severe traumatic brain injury: A meta-analysis with trial sequential analysis.* Aust Crit Care, 2020. **33**(4): p. 375-381.
27. Kim, J.H., et al., *Therapeutic Hypothermia in Critically Ill Patients: A Systematic Review and Meta-Analysis of High Quality Randomized Trials.* Crit Care Med, 2020. **48**(7): p. 1047-1054.
28. Wu, X., et al., *The effectiveness of early prophylactic hypothermia in adult patients with traumatic brain injury: A systematic review and meta-analysis.* Aust Crit Care, 2021. **34**(1): p. 83-91.
29. Du, Q., et al., *Effect of Hypothermia Therapy on Children with Traumatic Brain Injury: A Meta-Analysis of Randomized Controlled Trials.* Brain Sci, 2022. **12**(8).

30. Cumpston, M., et al., *Updated guidance for trusted systematic reviews: a new edition of the Cochrane Handbook for Systematic Reviews of Interventions.* Cochrane Database of Systematic Reviews, 2019(10).
31. Geng, M., et al., *Effects of therapeutic hypothermia on the safety of children with severe traumatic brain injury: a systematic review and meta-analysis.* Transl Pediatr, 2022. **11**(6): p. 909-919.
32. Utsumi, S., et al., *Targeted Temperature Management in Pediatric Traumatic Brain Injury: A Systematic Review and Network Meta-Analysis.* World Neurosurg, 2023. **173**: p. 158-166.e2.
33. Martyniuk, A., et al., *Therapeutic Hypothermia Compared with Normothermia in Adults with Traumatic Brain Injury; Functional Outcome, Mortality, and Adverse Effects: A Systematic Review and Meta-Analysis.* Neurocrit Care, 2024.

C. Results of the meta-analysis performed by the included studies

**Author/published year**	**Mortality**	**Outcome (poor outcome or favorable outcome)**	**Pneumonia**	**Other complications of hypothermia assessment?**
Harris et al. (2002) (1)	-	OR of TH on GOS score: 0.61 (0.26-1.46)	OR of pneumonia in TH: 2.05 (95% CI 0.79 – 5.32, p-value 0.14)	1) OR for arrhythmia 1.27 (0.38-4.25)<br><br>2) OR for an increase in prothrombin time of the TH group: 0.02 (-0.07 to 0.1)<br><br>3) OR for an increase in partial thromboplastin time of the TH group: 2.22 (1.73-2.71)
Henderson et al. (2003) (3)	OR of mortality in TH: 0.81 (0.59 - 1.13)	OR of TH on death or severe disability<br>: 0.75 (0.56 – 1.01)	OR of pneumonia in normothermia: 0.42 (0.25 – 0.7)	-
Mclntyre et al. (2003) (4)	1) RR of mortality in TH: 0.81 (0.69 – 0.96)<br><br>2) RR for mortality in the TH group for hypothermia >48h: 0.7 (0.56-0.87)	RR of poor neurologic outcome in TH: 0.78 (0.63-0.98)<br><br>2) RR for poor neurologic outcome in the TH group for hypothermia 24h: 0.61 (0.39-0.97)<br><br>3) RR for poor neurologic outcome in the TH group for hypothermia >48h: 0.65 (0.48-89)	-	-
Alderson et al. (2004) (5)	OR for mortality in TH: 0.8 (0.61 - 1.04)	OR for death or severe disability: 0.75 (0.56 – 1)	OR of pneumonia in TH: 1.95 (1.18 – 3.23)	-
Peterson et al. (2008) (6)	1) RR of mortality in the TH group: 0.8 (0.59-1.09)<br><br>2) RR of mortality in TH with hypothermia >48h duration:<br>0.51 (0.33-0.79)<br><br>3) RR of mortality in TH with hypothermia up to 48h:<br>1.03 (0.79-1.36)<br><br>4) RR of mortality in the TH group based on the data from studies that didn`t use barbiturates as an ICP lowering method: 0.58 (0.4-0.85)	1) RR of FN in the TH group: 1.25 (0.96-1.62)<br><br>2) RR of FN in TH with hypothermia >48h duration:<br>1.91 (1.28-2.85)<br><br>3) RR of FN in the TH group based on the data from studies that didn`t use barbiturates as an ICP lowering method: 1.79 (1.27-2.52)	1) RR of pneumonia in the TH group at the 12-month follow-up: 2.37 (1.37-4.10)<br><br>2) RR of pneumonia in the studies that used the barbiturate: 6.45 (2.47-16.85)	RR of arrhythmia in the TH group: 1.07 (0.53-2.18)

Sydenham et al. (2009) (7)	1) OR of mortality in the hypothermia group: 0.85 (0.68-1.06)<br><br>2) OR of mortality in hypothermia based on the studies with good allocation concealment methods: 1.11 (0.82-1.51)	1) OR of Unfavorable outcome in the hypothermia group at final follow-up: 0.77 (0.62-0.94)<br><br>2) OR of Unfavorable outcome in the hypothermia based on studies with good allocation concealment methods at final follow-up: 0.93 (0.7-1.23)	1) OR of pneumonia in TH group: 1.35 (0.95-1.91)<br><br>2) OR of pneumonia based on studies with good allocation concealment methods: 0.84 (0.52-1.35)	-
Fox et al. (8)	1) RR of prophylactic TH on mortality in the TH group: 0.73 (0.62–0.85)<br><br>2) RR of short-term prophylactic hypothermia on mortality: 0.98 (0.75-1.3)<br><br>3) RR for long-term prophylactic hypothermia on mortality: 0.62 (0.51 – 0.76)	1) RR for FN in TH group: 1.52 (1.28–1.80)<br><br>2) RR for FN in short-term hypothermia: 1.31 (0.94 – 1.83)<br><br>3) RR for FN in long-term hypothermia: 1.68 (1.44-1.96)	-	Hypokalemia was the most common electrolyte abnormality and was treated without life-threatening outcomes.
Georgiou et al. (2013) (10)	1) RR of prophylactic TH on mortality: 0.84 (0.72–0.98)<br><br>2) OR of high-quality studies on the effects of prophylactic TH on mortality: 1.28 (0.89 – 1.83)	1) RR of prophylactic TH on UFN: 0.81 (0.73 – 0.89)<br><br>1) RR of high-quality studies on the effects of prophylactic TH on UFN: 1.07 (0.92-1.24)	-	-
Ma et al. (2013) (11)	RR for mortality in the hypothermia group: 1.73 (1.06-2.84)	-	RR for pneumonia in the hypothermia group: 0.9 (0.73-1.12)	RR for cardiac arrhythmia: 2.57 (1.01-6.54)
Crossley et al. (2014) (12)	1) RR of death in the normothermia group: 1.31 (1.13 - 1.52)<br><br>2) RR of mortality in the normothermia group based on the data from the lower risk of bias studies: 1.62 (1.3-2.01)	1) RR for poor outcomes in the normothermia group: 1.49 (1.27-1.74)<br><br>2) RR of poor outcome in the normothermia group based on the data from the lower risk of bias studies: 1.67 (1.45-1.92)	1) RR for pneumonia in the TH group: 0.81 (0.62 -1.05)<br><br>2) RR for pneumonia in the TH group based on the studies with low risk of bias: 0.87 (0.71-1.07)	-
Geurts et al. (2014) (13)	-	-	-	The risk of any infection in the TH group was 1.31 (0.95-1.81)
Zhang et al. (2015) (16)	1) RR of mortality in the TH group: 1.84 (1.15-2.93)<br><br>2) RR of mortality in the TH group of severe TBI patients (GCS ≤8): 1.75 (1.09-2.81)	1) RR for FN in the TH group after 3-month follow-up: 0.89 (0.68-1.16)<br><br>2)RR for UFN in the TH after 3-month follow-up: 1.19 (0.8-1.76)	RR of pneumonia in the TH group: 0.84 (0.63-1.12)	1) RR of bleeding in the TH group: 0.94 (0.39-2.26)<br><br>2) RR of arrhythmia in the TH group: 2.6 (1.06-6.41)

		3) RR for FN in the TH group after 6-month follow-up: 0.91 (0.78-1.07)<br><br>2)RR for UFN in the TH after 6-month follow-up: 1.18 (0.88-1.59)		
Zhu et al. (2016) (17)	1) RR for mortality in the hypothermia group in the 3-month post-TBI: 0.95 (0.59-1.55)<br><br>2) RR for mortality in the hypothermia group in the 6-month post-TBI: 0.96 (0.76-1.23)<br><br>3) RR for mortality in the hypothermia group for mortality at the final follow-up based on the data from the low-risk-of-bias studies: 1.22 (0.97-1.54)	1) RR for UFN in the hypothermia group in the 3-month post-TBI: 0.79 (0.56-1.12)<br><br>2) RR for UFN in the hypothermia group in the 6-month post-TBI: 0.8 (0.63-1)<br><br>3) RR for mortality in the hypothermia group for UFN at the final follow-up based on the data from the low-risk-of-bias studies: 0.84 (0.62-1.15)	RR for pneumonia in the hypothermia group: 1.51 (1.12-2.03)	1) RR of cardiac complications in the hypothermia group: 1.75 (1.14-2.7)<br><br>2) RR of bleeding in the hypothermia group: 1.28 (0.4-4.15)
Zang et al. (2017) (20)	RR for mortality at the final follow-up in the TH group was 0.78 (0.64-0.96)	The TH group's RR for poor neurologic outcome was 0.71 (0.6-0.84)	-	-
Leng et al. (2018) (21)	OR of mortality in the hypothermia group: 1.21 (0.93 – 1.57)	1) OR of FN in the hypothermia group: 1 (0.8-1.26)<br><br>[the text claims to report RR, but the forest plot reports the OR]	-	-
Olah et al. (2018) (22)	1) OR of mortality between two groups: 0.675 (0.517-0.882)<br><br>2) OR of mortality between two groups in 32-33˚C hypothermia: 0.705 (0.378-1.317)<br><br>3) OR of mortality between two groups in 33-35˚C: 0.627 (0.393-1.001)<br><br>4) OR of mortality between >48h hypothermia and normothermia: 0.534 (0.392-0.729)<br><br>5) OR of mortality between 24-48h hypothermia and normothermia: 0.877 (0.649-1.185)	-	-	-

	6) OR of mortality between hypothermia with <0.25°C/h and normothermia: 0.575 (0.369-0.895)<br><br>7) OR of mortality between hypothermia with >0.25°C/h and normothermia: 0.742 (0.528-1.042)<br><br>8) OR of mortality between two groups in studies adjusted based on ICP: 0.53 (0.383-0.734)<br><br>9) OR of mortality in the studies not adjusted based on ICP: 0.849 (0.636-1.133)			
Watson et al. (2018) (23)	1) RR of mortality in the TH group: 0.83 (0.73-0.94)<br><br>2) RR of mortality in the TH group based on the studies with low risk of bias: 1.37 (1.04-1.79)<br><br>3) RR of mortality in the 3 months follow-up in the TH: 0.73 (0.46-1.17)<br><br>4) RR of mortality in the >6-month follow-up after the TBI: 0.84 (0.73-0.96)	1) RR of poor outcome in the TH group: 0.81 (0.75-0.87)<br><br>2) RR of poor outcome in the TH group based on the studies with low risk of bias: 1.16 (1.02-1.32)	RR of pneumonia in the TH group: 1.5 (1.23-1.83)	-
Chen et al. (2019) (24)	1) RR for mortality in the TH group: 0.91 (0.80 -1.03)<br><br>2) RR for mortality in TH use for treatment: 0.66 (0.49 – 0.88)<br><br>3) RR for mortality when TH is used for prevention: 1.12 (0.93 – 1.36)<br><br>4) RR for mortality in early TH: 0.83 (0.71-0.96)<br><br>5) RR for mortality in the control group compared to late TH: 1.12 (0.9-1.4)	RR for UFN: 0.78 (0.67 - 0.91)	RR for pneumonia in the TH group: 1.48 (1.11 – 1.97)	-

	6) RR of mortality in the TH group in studies with a low risk of bias: 1.31 (1.05-1.63)			
Huang et al. (2020) (26)	RR for mortality in the TH group was 0.94 (0.77-1.14)	1) RR of FN outcome in the TH group was: 1.2 (1.01-1.42)<br><br>2) RR for FN outcome at 3-month follow-up: 1 (0.57-1.76)<br><br>3) RR for FN outcome at 6-month follow-up: 1.06 (0.85-1.32)<br><br>4) RR for FN outcome at 12-month follow-up: 1.56 (1.2-2.03)<br><br>5) RR for mortality at 24-month follow-up: 1.79 (1.25-2.56) _	-	-
Kim et al. (2020) (27)	RR for mortality in the TH group: 1.26 (1.07-1.48)	-	-	-
Wu et al. (2021) (28)	1) RR of prophylactic hypothermia on mortality in the TH at the end of 6 months: 1.11 (0.9-1.37)<br><br>2) RR of prophylactic short-term hypothermia (≤48 h) on mortality in the TH at the end of 6 months: 1.04 (0.78-1.39)<br><br>3) RR of prophylactic long-term hypothermia (>48 h) mortality in the TH at the end of 6 months: 1.19 (0.88-1.6)	1) RR of FN in the prophylactic hypothermia group at the end of 6 months: 1.03 (0.91 -1.16)<br><br>2) RR of FN in the short-term (≤48 h) prophylactic hypothermia group at the end of 6 months: 1.1 (0.82-1.47)<br><br>3) RR of FN in the long-term (>48 h) prophylactic hypothermia group at the end of 6 months: 1.01 (0.8-1.28)	-	-
Du et al. (2022) (29)	RR of TH intervention on mortality: 1.57 (0.96-2.55)	The mean difference in GOS score was significantly higher in the hypothermia group (p-value: 0.01)	-	RR of incidence of complications: 0.98 (0.82-1.16)
Geng et al. (2022) (31)	OR of mortality in the hypothermia group: 1.72 (95%CI 0.98-3.02, p-value: 0.04)	-	-	1) OR of adverse outcomes in the hypothermia group: 1.39 (0.86-2.25)<br><br>2) OR of infection in the hypothermia group: 0.79 (0.51-1.23)<br><br>3) OR of arrhythmia in the hypothermia group: 3.10 (0.77-12.5)

Utsumi et al. (2023) (32)	RR of normothermia on mortality: 1.47 (0.69 – 3.13)	RR of TH on FN: 0.91 (0.79 – 1.04)	-	RR for arrhythmia in the TH group: 2.36 (0.74-7.46)
Martyniuk et al. (2024) (33)	1) RR for mortality in the TH group: 0.81 (0.68-0.96)<br><br>2) RR of mortality in the TH group based on the low-risk-of-bias studies: 1.14 (0.81-1.61)<br><br>3) RR of mortality in the TH group based on the data from patients who underwent a neurosurgical intervention: 0.57 (0.27-1.23)<br><br>4) RR of mortality in the TH based on studies with systemic surface cooling: 0.63 (0.53-0.75)<br><br>5) RR of mortality in the TH based on studies with systemic IV or gastric lavage cooling: 1.17 (0.99-1.38)<br><br>6) RR of mortality in the TH based on studies isolated cranial cooling: 0.76 (0.38-1.52)	1) RR of UFN in the TH group: 0.77 (0.67-0.88)<br><br>2) RR of UFN in the TH group based on the low-risk-of-bias studies: 0.89 (0.7-1.12)<br><br>3) RR of UFN in the TH group based on the data from patients who underwent a neurosurgical intervention: 0.79 (0.56-1.1)<br><br>4) RR of UFN in the TH based on studies with systemic surface cooling: 0.68 (0.59-0.79)<br><br>5) RR of UFN in the TH based on studies with systemic IV or gastric lavage cooling data: 1.17 (0.99-1.38)<br><br>6) RR of UFN in the TH based on studies isolated cranial cooling: 0.44 (0.29-0.67)	-	-

GCS: Glasgow Coma Scale, FN: favorable neurological outcome, OR: Odds ratio, RR: Relative risk, TBI: Traumatic brain injury, TH: therapeutic hypothermia, UFN: unfavorable neurological outcome

1. Harris OA, Colford JM, Jr., Good MC, Matz PG. The role of hypothermia in the management of severe brain injury: a meta-analysis. Arch Neurol. 2002;59(7):1077-83.
2. Jadad AR, Moore RA, Carroll D, Jenkinson C, Reynolds DJ, Gavaghan DJ, et al. Assessing the quality of reports of randomized clinical trials: is blinding necessary? Control Clin Trials. 1996;17(1):1-12.
3. Henderson WR, Dhingra VK, Chittock DR, Fenwick JC, Ronco JJ. Hypothermia in the management of traumatic brain injury. A systematic review and meta-analysis. Intensive Care Med. 2003;29(10):1637-44.
4. McIntyre LA, Fergusson DA, Hébert PC, Moher D, Hutchison JS. Prolonged therapeutic hypothermia after traumatic brain injury in adults: a systematic review. Jama. 2003;289(22):2992-9.
5. Alderson P, Gadkary C, Signorini DF. Therapeutic hypothermia for head injury. Cochrane Database Syst Rev. 2004(4):Cd001048.

6. Peterson K, Carson S, Carney N. Hypothermia treatment for traumatic brain injury: a systematic review and meta-analysis. J Neurotrauma. 2008;25(1):62-71.
7. Sydenham E, Roberts I, Alderson P. Hypothermia for traumatic head injury. Cochrane Database Syst Rev. 2009(2):Cd001048.
8. Fox JL, Vu EN, Doyle-Waters M, Brubacher JR, Abu-Laban R, Hu Z. Prophylactic hypothermia for traumatic brain injury: a quantitative systematic review. Cjem. 2010;12(4):355-64.
9. Harris B, Andrews PJ, Murray GD, Forbes J, Moseley O. Systematic review of head cooling in adults after traumatic brain injury and stroke. Health Technol Assess. 2012;16(45):1-175.
10. Georgiou AP, Manara AR. Role of therapeutic hypothermia in improving outcome after traumatic brain injury: a systematic review. Br J Anaesth. 2013;110(3):357-67.
11. Ma C, He X, Wang L, Wang B, Li Q, Jiang F, et al. Is therapeutic hypothermia beneficial for pediatric patients with traumatic brain injury? A meta-analysis. Child's Nerv Syst. 2013;29(6):979-84.
12. Crossley S, Reid J, McLatchie R, Hayton J, Clark C, MacDougall M, et al. A systematic review of therapeutic hypothermia for adult patients following traumatic brain injury. Crit Care. 2014;18(2):R75.
13. Geurts M, Macleod MR, Kollmar R, Kremer PH, van der Worp HB. Therapeutic hypothermia and the risk of infection: a systematic review and meta-analysis. Critical care medicine. 2014;42(2):231-42.
14. Higgins JPT, Altman DG, Gøtzsche PC, Jüni P, Moher D, Oxman AD, et al. The Cochrane Collaboration's tool for assessing risk of bias in randomised trials. BMJ. 2011;343:d5928.
15. Galvin IM, Levy R, Boyd JG, Day AG, Wallace MC. Cooling for cerebral protection during brain surgery. Cochrane Database Syst Rev. 2015;1(1):Cd006638.
16. Zhang BF, Wang J, Liu ZW, Zhao YL, Li DD, Huang TQ, et al. Meta-analysis of the efficacy and safety of therapeutic hypothermia in children with acute traumatic brain injury. World Neurosurg. 2015;83(4):567-73.
17. Zhu Y, Yin H, Zhang R, Ye X, Wei J. Therapeutic hypothermia versus normothermia in adult patients with traumatic brain injury: a meta-analysis. SpringerPlus. 2016;5(1).
18. Dunkley S, McLeod A. Therapeutic hypothermia in patients following traumatic brain injury: a systematic review. Nurs Crit Care. 2017;22(3):150-60.
19. Lewis SR, Evans DJ, Butler AR, Schofield-Robinson OJ, Alderson P. Hypothermia for traumatic brain injury. Cochrane Database Syst Rev. 2017;9(9):Cd001048.
20. Zang Z, Xu X, Xu S. The efficacy of therapeutic hypothermia in adult patients with traumatic brain injury: a systematic review and meta-analysis. Int J Clin Exp Med. 2017;10(6):8691-9.
21. Leng L. Hypothermia therapy after traumatic brain injury: A systematic review and meta-analysis. Turk Neurosurg. 2018;28(5):710-5.
22. Olah E, Poto L, Hegyi P, Szabo I, Hartmann P, Solymar M, et al. Therapeutic Whole-Body Hypothermia Reduces Death in Severe Traumatic Brain Injury if the Cooling Index Is Sufficiently High: Meta-Analyses of the Effect of Single Cooling Parameters and Their Integrated Measure. J Neurotrauma. 2018;35(20):2407-17.

23. Watson HI, Shepherd AA, Rhodes JKJ, Andrews PJD. Revisited: A Systematic Review of Therapeutic Hypothermia for Adult Patients Following Traumatic Brain Injury. Crit Care Med. 2018;46(6):972-9.
24. Chen H, Wu F, Yang P, Shao J, Chen Q, Zheng R. A meta-analysis of the effects of therapeutic hypothermia in adult patients with traumatic brain injury. Crit Care. 2019;23(1):396.
25. Meizikri R, Indiradini G. Induced hypothermia for traumatic brain injury: A systematic review. Indonesian Journal of Neurosurgery. 2019;2(1).
26. Huang HP, Zhao WJ, Pu J. Effect of mild hypothermia on prognosis of patients with severe traumatic brain injury: A meta-analysis with trial sequential analysis. Aust Crit Care. 2020;33(4):375-81.
27. Kim JH, Nagy Á, Putzu A, Belletti A, Biondi-Zoccai G, Likhvantsev VV, et al. Therapeutic Hypothermia in Critically Ill Patients: A Systematic Review and Meta-Analysis of High Quality Randomized Trials. Crit Care Med. 2020;48(7):1047-54.
28. Wu X, Tao Y, Marsons L, Dee P, Yu D, Guan Y, et al. The effectiveness of early prophylactic hypothermia in adult patients with traumatic brain injury: A systematic review and meta-analysis. Aust Crit Care. 2021;34(1):83-91.
29. Du Q, Liu Y, Chen X, Li K. Effect of Hypothermia Therapy on Children with Traumatic Brain Injury: A Meta-Analysis of Randomized Controlled Trials. Brain Sci. 2022;12(8).
30. Cumpston M, Li T, Page MJ, Chandler J, Welch VA, Higgins JPT, et al. Updated guidance for trusted systematic reviews: a new edition of the Cochrane Handbook for Systematic Reviews of Interventions. Cochrane Database of Systematic Reviews. 2019(10).
31. Geng M, Cui W, Cheng J, Li L, Cheng R, Wang X. Effects of therapeutic hypothermia on the safety of children with severe traumatic brain injury: a systematic review and meta-analysis. Transl Pediatr. 2022;11(6):909-19.
32. Utsumi S, Amagasa S, Yasuda H, Oishi T, Kashiura M, Moriya T. Targeted Temperature Management in Pediatric Traumatic Brain Injury: A Systematic Review and Network Meta-Analysis. World Neurosurg. 2023;173:158-66.e2.
33. Martyniuk A, Hart S, Lannon M, Mastrolonardo A, Kabbani A, Hafeez DA, et al. Therapeutic Hypothermia Compared with Normothermia in Adults with Traumatic Brain Injury; Functional Outcome, Mortality, and Adverse Effects: A Systematic Review and Meta-Analysis. Neurocrit Care. 2024.
