# Supplementary material for: Therapeutic hypothermia in patients with traumatic brain injury: an umbrella review
Source: BMC Neurol. 2025 Oct 24;25:440. doi: 10.1186/s12883-025-04463-3 (PMC12553179; doi:10.1186/s12883-025-04463-3)
Supplement: Supplementary file 3 — Supplementary Material 3. [file 12883_2025_4463_MOESM3_ESM.pdf]

A. Quality Assessment of the Included Studies

Author	Q1	Q2	Q3	Q4	Q5	Q6	Q7	Q8	Q9	Q10	Q11
**Lewis, 2017** [1]	Yes	Yes	Yes	Yes	Yes	Yes	Yes	Yes	Yes	Unclear	Yes
**Chen, 2019** [2]	Yes	Yes	Yes	Yes	Yes	Yes	Yes	Yes	Yes	Unclear	Yes
**Crossley, 2014 [3]**	Yes	Yes	Yes	Yes	Yes	Yes	Yes	Yes	Yes	Unclear	Yes
**Dunkley, 2016 [4]**	Yes	Yes	Unclear	Yes	Yes	Yes	N/A	N/A	N/A	Unclear	Yes
**Fox, 2010** [5]	Yes	Yes	Yes	Yes	Yes	Yes	Yes	Yes	Yes	Unclear	Yes
**Alderson, 2008 [6]**	Yes	Yes	Yes	Yes	Yes	Yes	Yes	Yes	Yes	Unclear	Yes
**Galvin, 2015** [7]	Yes	Yes	Yes	Yes	Yes	Yes	Yes	Yes	Yes	Unclear	Yes
**Georgiou, 2013 [8]**	Yes	Yes	Yes	Yes	Yes	Yes	Yes	Yes	Yes	Unclear	Yes
**Harris, 2002 [9]**	Yes	Yes	Unclear	Yes	Yes	Yes	Yes	Yes	Yes	Unclear	Yes
**Henderson, 2003 [10]**	Yes	Yes	Unclear	Yes	Yes	Yes	Yes	Yes	Yes	Unclear	Yes
**McIntyre, 2003 [11]**	Yes	Yes	Unclear	Yes	Yes	Yes	Yes	Yes	Yes	Unclear	Yes
**Utsumi, 2023 [12]**	Yes	Yes	Yes	Yes	Yes	Yes	Yes	Yes	Yes	Unclear	Yes
**Sydenham (2009)** [13]	Yes	Yes	Yes	Yes	Yes	Yes	Yes	Yes	Yes	Unclear	Yes
**Du (2022)** [14]	Yes	Yes	Unclear	Yes	Yes	Yes	Yes	Yes	Yes	Unclear	Yes
**Peterson, 2008 [15]**	Yes	Yes	Yes	Yes	Yes	Yes	Yes	Yes	Yes	Unclear	Yes
**Harris, 2012 [16]**	Yes	Yes	Yes	Yes	Yes	Yes	Yes	Yes	Yes	Yes	Yes
**Ma, 2013 [17]**	Yes	Yes	Yes	Yes	Yes	Yes	Yes	Yes	Yes	Unclear	Yes
**Geurts, 2014 [18]**	Yes	Yes	Yes	Yes	Yes	Yes	Yes	Yes	Yes	Unclear	Yes
**Zhang, 2015 [19]**	Yes	Yes	Yes	Yes	Yes	Yes	Yes	Yes	Yes	Unclear	Yes
**Zhu, 2016 [20]**	Yes	Yes	Yes	Yes	Yes	Yes	Yes	Yes	Yes	Unclear	Yes
**Zang, 2017 [21]**	Yes	Yes	Yes	Yes	Yes	Yes	Yes	Yes	Yes	Unclear	Yes

**Leng, 2018 [22]**	Yes	Yes	Unclear	Yes	Yes	Yes	Yes	Yes	Yes	Unclear	Yes
**Olah, 2018 [23]**	Yes	Yes	Yes	Yes	Yes	Yes	Yes	Yes	Yes	Unclear	Yes
**Watson, 2018 [24]**	Yes	Yes	Yes	Yes	Yes	Yes	Yes	Yes	No	Unclear	Yes
**Meizikri, 2019 [25]**	Yes	Yes	Yes	Yes	Yes	Yes	Yes	N/A	N/A	Unclear	Yes
**Huang, 2020 [26]**	Yes	Yes	Yes	Yes	Yes	Yes	Yes	Yes	Yes	Unclear	Yes
**Kim, 2020 [27]**	Yes	Yes	Yes	Yes	Yes	Yes	Yes	Yes	Yes	Unclear	Yes
**Wu, 2021 [28]**	Yes	Yes	Yes	Yes	Yes	Yes	Yes	Yes	No	Unclear	Yes
**Geng, 2022 [29]**	Yes	Yes	Yes	Yes	Yes	Yes	Yes	Yes	No	Unclear	Yes
**Martyniuk, 2024 [30]**	Yes	Yes	Yes	Yes	Yes	Yes	Yes	Yes	No	Unclear	Yes

Y = Yes, N = No, U = Unclear.

JBI critical appraisal checklist for systematic reviews and research syntheses: Q1 = Is the review question clearly and explicitly stated? Q2 = Were the inclusion criteria appropriate for the review question? Q3 = Was the search strategy appropriate? Q4 = Were the sources and resources used to search for studies adequate? Q5 = Were the criteria for appraising studies appropriate? Q6 = Was critical appraisal conducted by two or more reviewers independently? Q7 = Were there methods to minimize errors in data extraction? Q8 = Were the methods used to combine studies appropriate? Q9 = Was the likelihood of publication bias assessed? Q10 = Were recommendations for policy and/or practice supported by the reported data? Q11 = Were the specific directives for new research appropriate? <40%score = very low quality score; 40%-60% = low quality score; 60%-80% = moderate quality score; >80% = high quality score

1. Lewis, S.R., et al., *Hypothermia for traumatic brain injury.* Cochrane Database Syst Rev, 2017. **9**(9): p. Cd001048.
2. Chen, H., et al., *A meta-analysis of the effects of therapeutic hypothermia in adult patients with traumatic brain injury.* Crit Care, 2019. **23**(1): p. 396.
3. Crossley, S., et al., *A systematic review of therapeutic hypothermia for adult patients following traumatic brain injury.* Crit Care, 2014. **18**(2): p. R75.
4. Dunkley, S. and A. McLeod, *Therapeutic hypothermia in patients following traumatic brain injury: a systematic review.* Nurs Crit Care, 2017. **22**(3): p. 150-160.
5. Fox, J.L., et al., *Prophylactic hypothermia for traumatic brain injury: a quantitative systematic review.* Cjem, 2010. **12**(4): p. 355-64.
6. Alderson, P., C. Gadkary, and D.F. Signorini, *Therapeutic hypothermia for head injury.* Cochrane Database Syst Rev, 2004(4): p. Cd001048.
7. Galvin, I.M., et al., *Cooling for cerebral protection during brain surgery.* Cochrane Database Syst Rev, 2015. **1**(1): p. Cd006638.
8. Georgiou, A.P. and A.R. Manara, *Role of therapeutic hypothermia in improving outcome after traumatic brain injury: a systematic review.* Br J Anaesth, 2013. **110**(3): p. 357-67.
9. Harris, O.A., et al., *The role of hypothermia in the management of severe brain injury: a meta-analysis.* Arch Neurol, 2002. **59**(7): p. 1077-83.
10. Henderson, W.R., et al., *Hypothermia in the management of traumatic brain injury. A systematic review and meta-analysis.* Intensive Care Med, 2003. **29**(10): p. 1637-44.
11. McIntyre, L.A., et al., *Prolonged therapeutic hypothermia after traumatic brain injury in adults: a systematic review.* Jama, 2003. **289**(22): p. 2992-9.
12. Utsumi, S., et al., *Targeted Temperature Management in Pediatric Traumatic Brain Injury: A Systematic Review and Network Meta-Analysis.* World Neurosurg, 2023. **173**: p. 158-166.e2.
13. Sydenham, E., I. Roberts, and P. Alderson, *Hypothermia for traumatic head injury.* Cochrane Database Syst Rev, 2009(2): p. Cd001048.
14. Du, Q., et al., *Effect of Hypothermia Therapy on Children with Traumatic Brain Injury: A Meta-Analysis of Randomized Controlled Trials.* Brain Sci, 2022. **12**(8).
15. Peterson, K., S. Carson, and N. Carney, *Hypothermia treatment for traumatic brain injury: a systematic review and meta-analysis.* J Neurotrauma, 2008. **25**(1): p. 62-71.
16. Harris, B., et al., *Systematic review of head cooling in adults after traumatic brain injury and stroke.* Health Technol Assess, 2012. **16**(45): p. 1-175.
17. Ma, C., et al., *Is therapeutic hypothermia beneficial for pediatric patients with traumatic brain injury? A meta-analysis.* Childs Nerv Syst, 2013. **29**(6): p. 979-84.
18. Geurts, M., et al., *Therapeutic Hypothermia and the Risk of Infection: A Systematic Review and Meta-Analysis.* Critical Care Medicine, 2014. **42**(2): p. 231-242.
19. Zhang, B.F., et al., *Meta-analysis of the efficacy and safety of therapeutic hypothermia in children with acute traumatic brain injury.* World Neurosurg, 2015. **83**(4): p. 567-73.
20. Zhu, Y.F., et al., *Therapeutic hypothermia versus normothermia in adult patients with traumatic brain injury: a meta-analysis.* Springerplus, 2016. **5**.
21. Zang, Z.P., X.I. Xu, and S.M. Xu, *The efficacy of therapeutic hypothermia in adult patients with traumatic brain injury: a systematic review and meta-analysis.* International Journal of Clinical and Experimental Medicine, 2017. **10**(6): p. 8691-8699.
22. Leng, L., *Hypothermia Therapy after Traumatic Brain Injury: A Systematic Review and Meta-Analysis.* Turk Neurosurg, 2017.
23. Olah, E., et al., *Therapeutic whole-body hypothermia reduces death in severe traumatic brain injury if the cooling index is sufficiently high: meta-analyses of the effect of single ….* Journal of …, 2018.
24. Watson, H.I., et al., *Revisited: A Systematic Review of Therapeutic Hypothermia for Adult Patients Following Traumatic Brain Injury.* Crit Care Med, 2018. **46**(6): p. 972-979.
25. Meizikri, R. and G. Indiradini, *Induced hypothermia for traumatic brain injury: A systematic review.* Indonesian Journal of Neurosurgery, 2019. **2**(1).
26. Huang, H.-p., W.-j. Zhao, and J. Pu, *Effect of mild hypothermia on prognosis of patients with severe traumatic brain injury: A meta-analysis with trial sequential analysis.* Australian Critical Care, 2020. **33**(4): p. 375-381.
27. Kim, J.H., et al., *Therapeutic hypothermia in critically ill patients: a systematic review and meta-analysis of high quality randomized trials.* Critical Care …, 2020.
28. Wu, X., et al., *The effectiveness of early prophylactic hypothermia in adult patients with traumatic brain injury: A systematic review and meta-analysis.* Australian critical care : official journal of the Confederation of Australian Critical Care Nurses, 2020.
29. Geng, M., et al., *Effects of therapeutic hypothermia on the safety of children with severe traumatic brain injury: a systematic review and meta-analysis.* Transl Pediatr, 2022.
